# Physiochemical, Bio, Thermal, and Non-Thermal Processing of Major and Minor Millets: A Comprehensive Review on Antinutritional and Antioxidant Properties

**DOI:** 10.3390/foods13223684

**Published:** 2024-11-19

**Authors:** Suhan Bheemaiah Balyatanda, N. A. Nanje Gowda, Jeyamkondan Subbiah, Snehasis Chakraborty, P. V. Vara Prasad, Kaliramesh Siliveru

**Affiliations:** 1 Department of Grain Science & Industry, Kansas State University, Manhattan, KS 66506, USAsnehasis@ksu.edu (S.C.); 2 Department of Food Science, University of Arkansas Division of Agriculture, Fayetteville, AR 72207, USA; 3 Department of Agronomy, Kansas State University, Manhattan, KS 66506, USA; vara@ksu.edu

**Keywords:** tannins, phytates, phytochemicals, bioactive, phenols, antioxidants

## Abstract

Millets are recognized as future foods due to their abundant nutrition and resilience, increasing their value on the global stage. Millets possess a broad spectrum of nutrients, antinutrients, and antioxidants, making it imperative to understand the effects of various processing methods on these components. Antinutritional factors interfere with the digestibility of macro-nutrients and the bioavailability and bio accessibility of minerals. This necessitates methods to reduce or eliminate antinutrients while improving nutritive and antioxidant value in food. This review aims to elucidate the rationale behind processing choices by evaluating the scientific literature and examining the mechanisms of processing methods, categorized as physiochemical, bio, thermal, novel non-thermal, and their combination techniques. Physiochemical and bioprocessing methods alter antinutrients and antioxidant profiles through mass transfer, enzyme activation, product synthesis, microbial activity, and selective removal of grain layers. Thermal methods break functional bonds, modify the chemical or physical structures, enhance kinetics, or degrade heat-labile components. Non-thermal techniques preserve heat-sensitive antioxidants while reducing antinutrients through structural modifications, oxidation by ROS, and break down the covalent and non-covalent bonds, resulting in degradation of compounds. To maximize the trade-off between retention of beneficial components and reducing detrimental ones, exploring the synergy of combination techniques is crucial. Beyond mitigating antinutrients, these processing methods also stimulate the release of bioactive compounds, including phenolics, flavonoids, and peptides, which exhibit potent health-promoting properties. This review underscores the transformative potential of processing technologies in enhancing millets as functional ingredients in modern diets, promoting health and advancing sustainable food practices.

## 1. Introduction

Millets (Family: Poaceae) are known as “Nutri-cereals” due to their balanced macro- and micro-nutrient composition, contributing to the overall diet of humans. Millets are excellent sources of carbohydrates (60–75%), dietary fiber (6–12%), and proteins (7–13%). Millets also contain important minerals like iron, calcium, magnesium, phosphorus, and vitamins, especially B vitamins [1,2]. Millets contain health-promoting phytochemicals, including polyphenols, phytosterols, phytoestrogens, tannins, phenolic acids, tocopherols, and carotenoids [3,4]. The phytochemical compounds in millets offer several therapeutic values such as antioxidant [5,6], antimutagenic and anti-cancerous [7], antimicrobial [8], antidiabetic [9,10], anti-hypertensive, and cardio-protective [11,12] properties. Apart from various health benefits, these crops can thrive in diverse environmental conditions with low agricultural input. Millets are resilient to growing challenges, such as drought, heat, and unfavorable soil conditions, enabling successful cultivation in most soil conditions [13,14]. Therefore, millets play a crucial role in enhancing food security and combating malnutrition in many countries.

Millets have been consumed since ancient times across the world. They have played an instrumental role in human and cattle diets, particularly in Asian and African countries. Despite several benefits of millets, their production has remained relatively stable during the last few years. The world’s estimated production was about 29.62 million metric tons during 2023/2024 [15]. Africa, followed by India among Asian countries, are the top producing and consuming regions for millets. Various millet varieties, including finger (*Eleusine carocana*), pearl (*Pennisetum glaucum*), kodo (*Paspalum scrobiculatum*), foxtail (*Setaria italica*), proso (*Panicum miliaceum*) and little (*Panicum sumatrense*), barnyard (*Echinochloa frumentacea*), browntop (*Brachiaria ramose*), and sorghum (*Sorghum bicolor*), are cultivated in Asia [1]. The demand and popularity for millet-based food products are consistently increasing worldwide because of their gluten-free nature, low glycemic index (GI), and nutritional profile. Millets are recognized as a healthy alternative to traditional food grains such as rice, wheat, and corn. As a result, there has been a surge in the production and processing of millet to meet the growing demand [16]. In this context, a vast amount of research has been conducted to explore the health benefits of millets, including their antioxidant properties and free radicals scavenging ability. The processing and value addition to millets has evolved significantly to cater to the demand for diverse and convenient millet products. Conventional processing methods, such as milling, soaking, germination, and fermentation, are complemented with modern techniques like baking, extrusion, puffing, flaking, etc. The objective of processing methods is to improve the texture, taste, nutritional availability, and shelf life of millet-based products [17].

**Figure 1:**
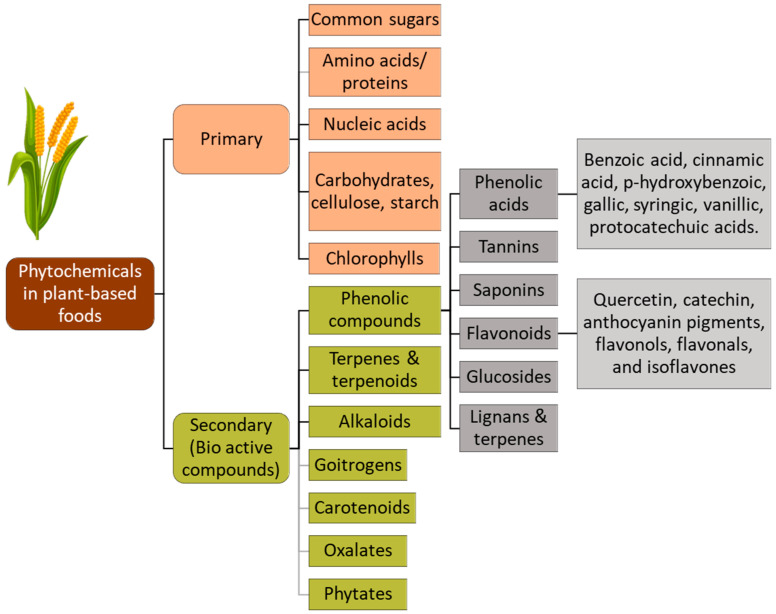
Major plant-based phytochemicals and antinutrients.

The biochemical profile of millets consists of compounds that function as antioxidants and antinutrients, presenting a nutritional complexity that may be beneficial or limiting, contingent upon the processing techniques applied. While millets possess phytochemicals that have positive impacts on health, they also contain antinutrients that may impede nutrient absorption and utilization [18]. The primary plant-based phytochemicals and antinutrients occurring in millets are shown in Figure 1. The phytochemicals termed antinutrients, such as phytates, tannins, saponins, and phenols, naturally occur in millets [19]. Antinutrients have a strong chelating ability to form complexes with monovalent and multivalent cations of minerals like potassium, calcium, zinc, iron, magnesium, etc., reducing bioavailability and limiting their absorption [20]. The antinutrients interfere with the starch and protein digestibility, resulting in the formation of protein-phenol complexes and metal chelation [21]. These antinutritional compounds in millets could potentially contribute to nutrient deficiencies when consumed in large quantities or with diets lacking other nutrient-rich foods. However, effective processing techniques, including thermal, physiochemical, bio, and novel non-thermal methods, have highlighted the potential to substantially reduce the antinutrients in millets, thus mitigating their negative impact on nutrient absorption and utilization [22,23,24]. For instance, a portion of antinutrient factors can be selectively removed by decortication (husk removal) and dehulling (bran removal) and by understanding the distribution of physiochemical processing components in the millet’s structure [25]. Similarly, soaking whole millet rice in water can leach a significant amount of tannins and phytates into the water, and germination degrades the phytates through the action of the phytase enzyme [26]. On the other hand, there is clinical evidence for the beneficial effects of compounds that are perceived as antinutrients. Tannins from plant sources have shown positive outcomes in reducing primary tumors of breast cancer through anticarcinogenic, anti-inflammatory, and antimutagenic manifestations and enhancement of blood pressure (proanthocyanidins) [27]. High molecular weight condensed tannins in millets, such as those in sorghum, demonstrate an increased affinity for binding with starch, which is directly proportional to its molecular weight. This interaction reduces the glycemic index, offering potential benefits to individuals with diabetes and obesity. Similarly, saponins have proved to exert a positive influence on the lipid and blood sugar profiles of patients with diabetes type II [28]. The coexistence of beneficial phytochemicals and antinutrients in millets emphasizes the significance of employing appropriate processing, cooking, and food preparation methods to maximize the health benefits of millets while minimizing any potential adverse effects [29]. Since antinutrients are biologically active secondary metabolites with both adverse effects and pharmacological significance, a thorough understanding of processing techniques is crucial before the selection of an appropriate method. Comprehending the role of processing techniques on the antioxidant and antinutritional properties of phytochemicals found in millets is critical to maximizing the nutritional quality and health benefits of millet-based foods. Employing appropriate processing techniques can preserve and enhance the antioxidant content, maximizing their protective effects against oxidative stress and chronic diseases. Additionally, effective processing methods must significantly reduce antinutritional factors, leading to improved nutrient bioavailability and minimizing potential negative effects [30]. Based on the method of processing, the processing techniques can be grouped as follows: (1) Thermal processing, including steaming, boiling, parboiling, roasting, flaking, extrusion, cooking, and microwaving, etc.; (2) physiochemical and bioprocessing techniques, including germination, fermentation, enzymatic, decortication, dehulling, milling, soaking, and malting; (3) non-thermal technologies, including application of irradiation, pulsed light, UV, ozone, cold plasma, high-pressure processing, pulsed electric field, ultrasound, etc.; and (4) combination techniques that synergistically reduce antinutritional factors (ANFs). 

The focus on processing techniques holds great potential for enhancing the overall nutritional quality and health-promoting properties and increasing the consumer acceptability of millet-based products. To the authors’ knowledge, to date, there have been limited comprehensive review articles focused on comprehending the effect of processing techniques on the reduction in antinutrients in millets and enhancing antioxidant value. The effect of processing techniques on nutritional properties in various minor and major millets has been extensively discussed and published by several researchers. Therefore, this review aims to comprehend the influence of different thermal, physiochemical, bioprocessing, and non-thermal techniques on the antinutritional and antioxidant properties of major and minor millets. This article also highlights the potential for incorporating millets into various food products to leverage their health-beneficial attributes. This article could serve as a guide by offering insights into the development of millet-based functional foods, promoting innovation in food processing and utilization of millets in diverse products, thus contributing to sustainable food systems and global food security. 

## 2. Search Strategy for the Articles

Various databases and search engines, including Google Scholar, PubMed, and Scopus, were used to search for relevant research articles, review articles, and book chapters. The search employed specific keywords related to “Processing-pearl millet”, “Processing and foxtail millets”, “Processing and proso millets”, “Processing and little millet”, “Processing and kodo millet”, “millets and phytochemicals”, “millets and antioxidant”, “millets and antinutritional”, “millets” OR “antinutrients” OR “phytochemicals” OR “phenols” OR “tannin” OR “phytate” OR “antioxidant” OR “nutria-cereals” OR “bioprocessing” OR “hydrothermal processing”, “cold plasma” AND “millets”, “irradiation AND millets”, “ultrasound AND soaking” OR “millets AND fermentation OR germination”, “nonthermal”, and “physiochemical”. The articles included in the review spanned from 2018 to May 2024. Back referencing of retrieved articles was done to search for additional relevant articles. However, due to limited literature availability, specifically on microwave processing in millets, the search was extended back to 2015 to ensure an adequate pool of relevant studies. In this review, a total of 2386 records were initially identified through a comprehensive query string. A filter was applied based on keywords, which reduced the number of records to 1231. After screening of articles based on title, about 356 records were retained, and further abstract screening resulted in 246 studies. Finally, about 179 studies were included in the narrative synthesis, ensuring sufficient articles for a comprehensive review study. 

## 3. Overview of Antinutritional and Antioxidant Properties of Phytochemicals Found in Selected Millets 

**Figure 2:**
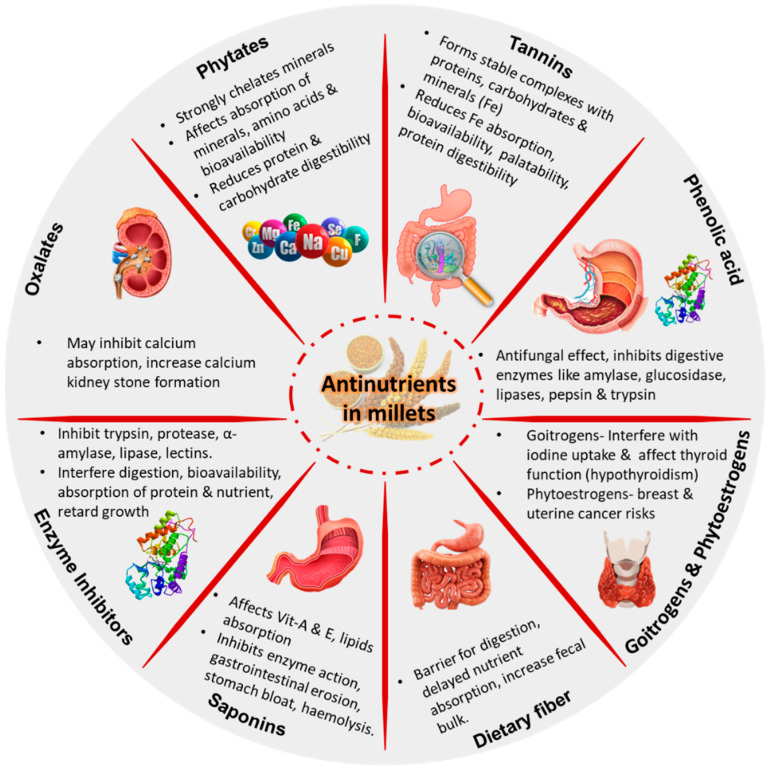
Potential health effects of common antinutrients in millets (Source: multiple citations of this article).

While the susceptibility to antinutrients varies among individuals, it is recommended to minimize the amount through processing [31]. Phytates, tannins, lectins, oxalates, and other antinutrients are frequently encountered in plant-based diets [32]. The various drawbacks and health issues that are frequently reported due to the excess consumption of antinutrients from plant sources are summarized in Figure 2.

Nevertheless, millets contain abundant phytochemical compounds that offer antioxidant properties, including phenolic compounds like flavonoids, tannins, phenolic acids, carotenoids, and tocopherols [32]. These compounds are crucial in enhancing nutritional value, building immunity, and serving as defense mechanisms by scavenging free radicals, countering oxidative stress, mitigating cellular damage, minimizing vegetative loss to the plant by deterring herbivores through raphides (oxalates), and inhibiting phytopathogens (saponins) [33]. The anti-nutritional and antioxidant properties of various phytochemical compounds of different millets are summarized in Table 1. For several millet varieties, the total phenolic content (TPC) ranges between 0.22 and 4.44 mg GAE/g of dry matter (dm). The kodo millet contains the highest levels of free and bond phenolics, indicating potential antioxidant properties. The kodo millet also exhibited notable alpha-amylase (125.8 µg GAE/mL) and alpha-glucosidase (92.6 µg GAE/mL) inhibitory activity, indicating its ability to reduce starch digestibility, which could be beneficial in regulating obesity and diabetes [34,35,36]. The highest DPPH radical scavenging activity (53.1–56.1%) in kodo compared to other millet varieties (foxtail, little, barnyard, proso) indicates its strong antioxidant capacity. In addition, the kodo millet includes the lowest concentration of antinutrients such as tannins (0.48 mg/g; d.w), phytic acid (1.2–8.3 mg/g; d.w), and oxalates (0.03 mg/g; d.w). Table 2 summarizes the concentrations of several phytochemicals, antinutrients, and antioxidants in various millets (whole grains and processed flour), as reported in published research. The abundant phytochemicals and antioxidants witnessed in various millet varieties make them nutritionally superior alternatives to common staple cereal crops. 

## 4. Effect of Processing on Antinutrients and Antioxidant Properties of Millets 

This review consolidates studies on thermal, physiochemical, bio, and non-thermal methods employed in millets, their processing mechanisms, and subsequent effects on the antioxidants and antinutrients. Table 3 and Table 4 illustrate the impact of the applied process on the concentration of antinutrients and antioxidants, indicating whether the concentrations increased, decreased, or remained unchanged. The summary of the results of physiochemical, bio, thermal, and non-thermal processing methods on millets, their influences on antinutritional compounds, and the inference for the respective changes have been elucidated in Table 5, Table 6 and Table 7, respectively.

### 4.1. Effect of Processing on Polyphenols 

Phenolic compounds in millets contain phenolic acids and flavonoids and are recognized for their potential natural antioxidants in food and biological systems. While phenolic acids (hydroxycinnamic acid and hydroxybenzoic acid) occur in the bound state, flavonoids (quercetin, apigenin, taxifolin, catechin) are found in the free state. Millet phenols are rich in phenolic acids that effectively prevent aging and cancer initiation and progression in vitro [122]. The radical intermediates of polyphenols prevent the oxidation of lipids and other food ingredients. Flavonoids, a class of phenolic acids, are known to exhibit antimicrobial, antibiotic, and anti-inflammatory properties. Phenolics are distributed unevenly throughout the grain, while concentrated in the seed coat. The choice of processing method adopted substantially influences the final concentration. Consumption of millets is often preceded by physiochemical, bio, thermal, or non-thermal processing or their combinations to improve palatability and reduce antinutrients. They often alleviate the phenolic profile or reduce their availability and consequently the flavor of millets. It is crucial to minimize the loss of bioactive compounds while also achieving substantial reductions in antinutrient content.

#### 4.1.1. Effect of Physiochemical and Bioprocessing on Polyphenols 

Soaking is a fundamental processing method that includes the diffusion of water into grains through the hilum until an equilibrium moisture content is reached. This process often facilitates dehulling, milling, germination, cooking, and enzyme activation. For example, in pearl millet grains soaked overnight (12 h), a reduction in polyphenolic content from 241.47 to 184.43 GAE mg/100 g was recorded [93]. Similar findings were noted during soaking finger millet at 30, 40, and 50 °C for 0 to 24 h at 6 h intervals [79]. However, the Total Flavonoid Content (TFC) increased from 66.3 to 103.9 mg/100 g; dry weight and the DPPH free radical scavenging activity (FRSA) increased by 1 to 4% after soaking. The decrease in TPC during soaking is due to the activation of the polyphenol oxidase enzyme, which causes polyphenol degradation. While the soaking temperature did not have a discernible effect on TPC, increasing the soaking time significantly reduced the TPC due to increased water absorption, which facilitates enzyme catalysis.

Germination refers to the development of the embryonic axis in a dormant seed when hydrated under optimal oxygen and temperature conditions. The metabolic activity of the grain increases upon germination, leading to enhanced enzyme activity and metabolic products. Studies have reported that the phenolic level in proso millet increased up to 3.5 times after germination for 5 days [93]. Soaking foxtail and proso millet for 15 h and germinating them at 24 °C for 4 days doubled the phenolic acids and flavonoids in both millets [84]. Similarly, germination significantly increased TPC, TFC, and proanthocyanidins (PAC) in barnyard, foxtail, and kodo millet. The TPC increase during germination is a direct result of the biosynthesis of phenolic compounds or the action of glucosidase enzymes [91]. Soaking time and germination temperature are two critical factors that influence TPC. For example, foxtail millet was germinated for 19.5 to 46.45 h, with soaking times ranging from 5.27 to 18.73 h and germination temperatures varying from 13.18 °C to 46.8 °C. The TPC, free phenols, and bound phenols in defatted flour increased from 33.17 to 57.17 GAE/100 g (dw), 9.79 to 21.75 mg (GAE)/100 g (dw), and 23.38 to 35.42 mg (GAE)/100 g (dw) of sample, respectively. TFC including free and bound flavonoids followed a similar trend, increasing from 28.1 to 57.72 mg RU/g in plant extract (dw), 2.43 to 25.74 mg RU/g (dw), and from 15.67 to 31.98 mg RU/g (dw), respectively [86].

Another study on foxtail and little millets, germinated at 35 °C and 30 °C for 24 h, observed an increase in TPC (mg GAE/100 g) of whole foxtail from 55.9 to 69.2, while it slightly decreased from 132 to 123.3 in finger millet after germination. An increase in TFC (mg RE/g) was also observed in whole foxtail millet (12 to 12.4) and little millet (7.4 to 10) after germination [5]. Prolonged soaking time provides sufficient water for metabolic enzymes to maintain their conformational structure and catalyze reactions. The enzymes (cell wall degrading) release the free, bound, and total phenolic compounds during germination. Meanwhile, the TFC, which constitutes free phenols, increases as a result of biosynthesis and tannin degradation. Contrary to the observed results, germination treatment (ambient temperature) of finger millet flour pre-soaked (9 h) and germinated at intervals of 0, 12, 24, 48, 72, and 96 h reduced TPC from 38.4% to 42.6%. However, flavonoid content and antioxidant activity (AOA) increased by 26.6% to 33.3% and 48.3% to 51.1%, respectively [40]. While most studies report enhanced TPC on germination [31,123,124], some studies also indicated a reduction in TPC. This reduction could be attributed to shorter germination times, which do not efficiently release the bound phenols, as observed in a study on hulled oats [125].

A study [126] reported that spontaneous fermentation of pearl millet at varying temperatures (20, 25, and 30 °C) for 72 h decreased the phytic acid levels and increased polyphenols. Additionally, fermentation for 10 days with *Rhizopus azygosporus* increased TPC from 6.6 to 21.8 mg GAE/g (dry weight). The antioxidant activity was 16.4 times higher in fermented pearl millet compared to unfermented samples. These results are due to the hydrolyzing effect of enzymes produced during fermentation, liberating conjugated phenols. In an attempt to valorize acid whey from the Greek yogurt industry and enhance its potential as a complementary food, a study [88] incorporated kodo and proso millet into acid whey at various concentrations (25% to 100%). The TPC (mg GAE/g) increased in kodo (0.09 to 0.15) and proso millet (0.12 to 0.17) with increased millet proportion (25% to 100%). The highest total phenol content at 100% concentration was 0.157 mg GAE/g for kodo millet and 0.168 mg GAE/g for proso millet, demonstrating that Lactic acid bacteria (LAB) metabolic enzymes could liberate the phenols in millets and improve phenolic and flavor profile. A study [82] observed the decrease in total phenols by 18.22% (7.41–6.06 mg GAE/g) and an increase in antioxidant activity by 83.10% (42.25–77.26%) with increased fermentation time (0 to 48 h) using fermented cabbage extract that supplements *L. plantarum* and *L. casei*. The β-glucosidase enzyme from LAB hydrolyzes phenolic glycosides, releasing free phenolic compounds and exhibiting high antioxidant activity.

The yeast fermentation of pearl millet flour was conducted with varying temperatures (30–45 °C), yeast concentrations (2–4%), and fermentation times (18–24 h) to assess their impact on TPC. The optimized condition included 2% yeast, 30 °C, and 18 h of fermentation. Across various treatments, TPC ranged from 32.74 to 46.43 µg GAE/mg (dry Weight (dw), and TFC ranged from 2.24 to 9.46 µg CAE/mg (dw). The highest TPC was achieved with a low yeast concentration (2%), 18 h of fermentation, and a high temperature (45 °C). The pH variation, enzymes, and high temperatures during fermentation enhanced the release of phenols [75]. Additionally, fermenting millet for 72 h with yeast and *Lactobacilli* resulted in a 36% increase in phenolic content [93]. Flour made from the light and dark brown finger millet varieties, increased the TPC from 166 to 488 mg/100 g (dw) and 420 to 606 mg/100 g (dw), respectively after spontaneous fermentation for 96 h. Similarly, TFC increased from 160–330 mg/100 g (dw) and 249–466 mg/100 g (dw), respectively. Contrarily, the total anthocyanin content significantly reduced from 58 to 32 mg of cyanidin-3-glucoside/100 g (dw) (light brown) and 69 to 38 mg of cyanidin-3-glucoside/100 g (dw) (dark brown) with increased fermentation duration.

The increase in TPC and TFC was because of the formation of enzymes such as amylase, glucosidase, phytase, tannase, lipase, cellulase, and inulinase during fermentation, which break down the cell wall and facilitate the release of flavonoids and free phenolics. However, the reduction in TAC was due to the action of glycosidase, which hydrolyses anthocyanins in the cotyledon [72]. A study [77] demonstrated that sorghum and pearl millet, when subjected to natural fermentation at 28 °C for 2–5 days and fermented for 0–96 h, enhanced TPC, TFC, and antioxidant activity (AOA). However, increasing the sprouting time linearly decreased TPC (21.1–16.25 mg/100 g; d.w) and linearly increased TFC (6.3–7.8 mg/100 g; d.w), FRAP expressed as ascorbic acid equivalent (29.6–32.1 mM/100 g), and DPPH Radical scavenging activity (RSA) (34.6–41.0 mM/100 g). Conversely, increased fermentation time (0–96 h) linearly increased the TPC (20.1–25.6 mg/100 g), TFC (6.3–8 mg/100 g), FRAP (29.6–31.4 mM/100 g), and DPPH FSA (34.6–38.8 mM/100 g).

The effect of LAB and yeast fermentation on foxtail millet was investigated in a separate study [48], which utilized *S. cerevisiae* in combination with ammonium sulphate (a fermentation activator), *L. brevis*, *L. planatarum,* and a combination of *L. brevis* and *S. cerevisiae* over fermentation periods of 12, 24, and 36 h. All treatments enhanced the TPC, with the highest value of 2.06 mg GAE/g for fermentation using *S. cerevisiae* with ammonium sulphate for 36 h. Similar findings were reported in finger millet flour, where the TPC of raw finger millet flour was enhanced from 1.07 to 2.01 mg GAE/g after fermentation with *S. cerevisiae* with ammonium sulphate for 36 h. It was concluded that an increase in antioxidant activity and total phenolic content was attributed to prolonged fermentation time.

Pearl millet fermented at 38 °C for various durations ranging from 0 to 96 h using a mixture of *L. pentosus*, *L. sanfranciscensis*, and yeast strains increased free phenols initially from 126.9–145.3 mg/100 g (dw) at 72 h fermentation and remained unaltered after 96 h. The microbial action during fermentation released phenolic compounds from soluble fibre, increasing the total phenols by 36% [71]. Further studies on fermentation were conducted using three microbial combinations containing yeast and LAB: FPM1 (fermented pearl millet with *S. boulardi*), FPM2 (fermented pearl millet with *S. cerevisiae + C. paralimentarius*), and FPM3 (fermented pearl millet with *H. uvarum + F. sanfranciscensis*). These were analyzed simultaneously with unfermented pearl, finger, and foxtail millet to assess their phenolic composition. Pearl millet exhibited the highest free phenols (0.3–0.74 mg/g; d.w), followed by finger (0.3 mg/g; d.w) and foxtail (0.26 mg/g; d.w) millets. Total phenols in unfermented pearl millet (2.26 mg/g; d.w by acid hydrolysis) were reduced in FPM1 (1.2 mg/g; d.w) but increased in FPM2 (2.74 mg/g; d.w) and FPM3 (2.57 mg/g; d.w) in pearl millet samples. It was observed that fermentation with a single culture or a mixture of cultures reduced the free phenolics. However, total phenols increased by acid hydrolysis when a mixture of yeast and LAB was used, unlike the significant reduction in TPC observed with single cultures [65]. Contrasting with previous findings, natural fermentation (72 h at 28 °C) of finger millet using LAB species (*B. cereus*, *L. plantarum*, *L. casei*, and *L. brevis*) reduced total phenols from 40.2 to 11.6 mg/100 g; d.w. This variation could be attributed to factors such as grain type, microbial species, pH, temperature, and time [73]. Steeping (28 °C for 16 h), fermentation (72 h), and germination (12 h) of pearl and finger millet noted a decrease in TPC by 14, 43, and 57% in pearl millet samples. Similarly, a decrease in TPC by 36% for germination and 26–29% fermentation was recorded in finger millet. In both samples, combination treatments reduced the TPC effectively [80].

A similar study on sorghum involved three wet processing methods: steeping for 16 h, germination (12 h of steeping followed by 3 days of sprouting at 30 °C), and natural fermentation for 72 h. Steeping reduced TPC by 15%, 55% in germination, and 23% in fermentation [127]. Most literature cited a positive correlation between TPC, fermentation time, temperature, and strain heterogeneity. A diverse microbial population contributed to a wide range of enzymes capable of liberating phenolic compounds, which is enhanced with adequate time and temperature. In contrast, studies on millet phenolics have reported a decrease in TPC with increased fermentation time, particularly when combined with treatments such as germination and steeping. These reductions are influenced by various factors, such as duration, temperature, microbial strain, pretreatment methods, and type of grain [26,65,79,80].

In a study [66], the development of beverages from sorghum grains and finger millet was analyzed by varying the malting duration (3, 4, and 5 days) and fermentation temperature (20 °C, 25 °C, 30 °C, and 35 °C). The increase in malting days from 3 to 5 resulted in a TPC of 0.92 GAE/100 g, while an elevated temperature of 35 °C raised the TPC to 1.66 GAE/100 g. Similarly, pearl millet malting through steeping (24 h at 30 °C), germinating (34 °C for 24 h), sprouting (48 h), drying (60 °C for 5–6 h), and disc milling enhanced phenolic content (38.36 mg/100 g) as compared to okra and wheat flour [68]. Contrary to these findings, a study [78] reported a 50% decrease in phenols and 41–84% decrease in flavonoids after 24 h and 48 h malting, with the grains steeped for 12–16 h, germinated for 24–36 h, and shade dried at 35 °C and 48 °C for 48 h in finger millet. The increase in free soluble phenols in the literature is attributed to the hydrolysis of bound phenols during steeping [61,66,68]. However, another study reported a decrease in TPC, attributed to malting activating endogenous enzymes that hydrolyze bound phenols, leading to their subsequent leaching during steeping and germination [78]. Foxtail and proso millets subjected to dehusking and debranning led to a decrease in TPC from 1.7 to 0.5 mg of CE/g of defatted flour (dw) and TFC from 0.7 to 0.14 mg of CE/g of defatted flour (dw). The decrease was attributed to the removal of outer bran layers and husk during successive processing steps, which are rich in phenolic compounds [38]. In agreement, another study [90] found that whole grains exhibited the highest TPC of 164.46 mg GAE/100 g of dry weight, followed by dehusked grains (145.81 mg GAE/100 g; d.w) and polished grains (69.37 mg GAE/100 g; d.w). The TFC results were exactly similar to that of TPC. The above fermentation studies demonstrated that the TPC and antioxidant activity in various millets are enhanced, however, the effects vary with the microbial culture, fermentation duration, and temperature. It is also evident that these enhancements are particularly associated with specific strains of yeast and *Lactobacillus*, although the results can differ due to microbial species, fermentation conditions, and grain types. Conversely, some fermentation and processing methods led to reductions in TPC, highlighting the complexity and variability in optimizing fermentation conditions for phenolic enrichment.

The physiochemical and bioprocessing methods highlight the influence of processing on the nutritional profile of millets, with a particular emphasis on antioxidant activity and reduction in antinutrients. Both germination and fermentation play a crucial role in enhancing phenolic and flavonoid contents through enzymatic activity, thereby boosting the antioxidant properties. Conversely, dehusking and debranning selectively remove several bioactive compounds (for example, phenolics) accumulated in outer layers. Research studies highlighted that factors such as millet varieties, processing techniques, and their conditions have a significant influence on TPC and TFC. This review elucidates the dynamic nature of physiochemical and bioprocessing in optimizing nutritional availability and enhancing health benefits through the reduction of ANF. Future research should investigate developing customized approaches to exploit these methods for sustainable food processing and enhanced dietary health outcomes.

#### 4.1.2. Effect of Thermal Processing on Polyphenols

The studies on the effect of thermal processing techniques on phenolic compounds reveal a significant enhancement in both free and bound phenolic contents, particularly in parboiling. There was a notable increase in the TPC of pearl and proso millet-based porridge; the free and bound phenolic contents were increased by 200% and 41%, respectively. In couscous, the free and bound phenolic contents enhanced by 64% and 47%, respectively. Similarly, parboiling enhanced the total free phenolics by 480% and 165%, and bound phenolics by 34% and 20% in proso millet porridge and couscous, respectively. The study also demonstrated the increase in millet yield and alteration in phenolic acid profile and digestibility in the end products of the parboiled millet [90]. The release of bound phenolics was attributed to the breakdown of cellular components of pearl millet during boiling for 10 min, which increased the phenolic content from 2394 to 3137 g GAE/g of dry weight [93]. Another similar study [98] showed that the millet products demonstrated higher free and bound phenolic contents and DPPH radical scavenging activity after partial or semi-boiling treatments. These studies highlight that heat-based processing techniques significantly enhance free and bound phenolic contents, resulting in higher antioxidant activity and yield. Thermal processes also alter the phenolic acid profile and influence the digestibility of end products. This is because the cell walls break down, and cellular components release bound phenolic compounds into a free form. This process increases the overall phenolic content and antioxidant activity. Additionally, heat treatment can alter the phenolic acid profile by converting certain phenolic compounds into more bioactive forms and improve digestibility by softening the millet matrix, making phenolics more accessible during digestion.

A study exploring the effects of wet heat processing, including steaming, pressure cooking, dry roasting, and open boiling of finger millet, revealed significant variations in phenolic content in final products. For example, pressure cooking increased soluble phenolics by 33%, open boiling by 67%, and dry roasting by 17%; boiling reduced soluble phenolics by 12–19%. The TPC of soluble phenolics ranged from 29.6 to 79.6 µM FAE/g, while that of bound phenolics ranged from 2.2 to 11.8 µM ferulic acid equivalents FAE/g. Soluble flavonoids (µM CE/g) decreased by 26%, 23%, and 13% with pressure cooking, roasting, and steaming, respectively, but increased by 0.5 to 1.5 times with open boiling. Open boiling retained most phenolics and antioxidant activity [92]. The phenolic content of pearl millet samples (5.56 mg GAE/g) decreased to 4.98 mg GAE/g after hydrothermal treatment (soaked for 1 h, ground, steamed at 100 °C for 5 min), while it increased to 6.06 mg GAE/g and 6.30 mg GAE/g for microwave (750 W, 2450 Hz for 90 s) and infrared (150 W, 0.7–1 µm wavelength at 70–80 °C for 5 min) treatments, respectively. The hydrothermal treatment was found to be a promising method with enhanced nutrient value, reduced glycemic index, and reduced bioaccessibility of phenolics by 10.6% [24]. Another study [128] investigated the hydrothermal processing of foxtail millet, utilizing either the soak-boil or soak-steam method. In the soak-boil method, foxtail millet was soaked for 2 h at 50.5 °C and boiled for 6.2 min, yielding a TPC of 45.19 mg GAE/100 g. On the other hand, the soak-steam method (soaking for 2.05 h at 44 °C, steaming at 110 °C for 5 min) resulted in a TPC of 48.64 mg GAE/100 g. Dry heat treatment increased TPC by liberating bound phenols. In contrast, hydrothermal treatment caused a reduction in phenols due to high temperatures or their leaching out and subsequent formation of protein complexes. A similar reduction in TPC (1.29 to 1.19 mg GAE/g) and DPPH free radical scavenging activity (FRSA) (38.5% to 35.6%) was observed in pearl millet samples blanched at 98 °C for 30 °C [49]. The above studies suggest that open boiling and microwave treatments enhance the phenolic content and antioxidant potential through breakdown of cell walls and release of bound phenolics, increasing the free phenolics availability. Conversely, hydrothermal treatments can reduce phenolic content and bioaccessibility due to high temperatures causing degradation or the formation of complexes with proteins, which decreases their availability. Dry heat treatments, like roasting, increase phenolic content by liberating bound phenolics through thermal breakdown. The method’s impact of processing on phenolic content and antioxidant activity depends on the treatment’s conditions to release or degrade phenolic compounds.

Thermal processing of barnyard, foxtail, and proso millets by steam and microwave treatments increased the TPC (mg FAE/100) in foxtail millet (from 72.7: raw to 76.22: steamed; 104.46: microwaved) and proso (from 74.43: raw to 79.5: steamed; 87.43: microwaved). However, for barnyard millet, TPC decreased from 80.14: raw to 76.08: steamed and to 73.65: microwaved [91]. The kodo millets were microwaved at 360, 540, and 720 W for 150, 210, and 270 s at 2450 MHz. The TPC and DPPH-AOA of the untreated kodo millet were measured at 3.26 mg GAE/100 g of dry weight and 92.1%, respectively. The microwave treatment increased the TPC at power levels and durations, ranging from 3.29 to 3.42 mg GAE/100 g; d.w at 360 W, 3.39 to 3.46 mg GAE/100 g; d.w at 540 W, and 3.53 to 4.23 mg GAE/100 g; d.w at 720 W, as the duration extended from 150 to 270 s. Conversely, the antioxidant activity declined from 91.1 to 87.9% with increased power levels and treatment durations [97]. The sorghum was overnight soaked, microwaved (450 and 700 W for 15 to 30 s), and subsequently germinated at 25 °C for 48 h. The optimized treatment (700 W for 15 s) offered the highest TFC of 1.05 mg QE/g, TPC of 1.27 mg GAE/g of dry weight (dw), DPPH antioxidant (AOX) of 89.7%, and FRAP AOX of 0.03 mM FE/g; d.w, compared to an untreated sample. Similarly, pearl millets were microwaved (900 W for 40 to 100 s) and pressure steamed (soak grains at 65 °C for 2 h) for 4, 6, 8, and 10 min. The TPC of untreated millets decreased linearly from 234.4 to 175.5 mg GAE/100 g of dry weight with increased microwaving duration (0–100 s). Additionally, the TPC of untreated millets decreased from 234.4 to 148 mg GAE/100 g with increasing steaming duration from 0–10 min [102]. Another study [4] observed that roasted millet exhibited higher antioxidant activity compared to steamed millet; this was because of the thermal degradation of cellular components, which improved the extraction of bound phenolics. Thermal treatments, including steaming and microwaving, were noticed to enhance the concentration of phenolic compounds in proso and foxtail millet. Additionally, the extrusion process of proso millet, characterized by low moisture levels and high screw speed, has successfully produced ready-to-eat products with enhanced antioxidant properties. A significant reduction in the TPC of pearl millet (2.58 mg/g) was recorded for pressure cooking (29%), open pan boiling (12–29%), and microwave heating (25–32%). However, sprouting had an insignificant effect on polyphenol concentration, while roasting enhanced polyphenols by 12% due to increased tannin content. Furthermore, bio-accessible polyphenol was improved by about 20% after roasting and sprouting processes [129].

Roasting proso millet at 110 °C for 10 min increased the TPC from 295 to 670 mg/100 g due to the increased hydrolysis of C-glycosyl flavones, leading to the release of phenolic compounds. In contrast, roasting pearl millet decreased the TPC from 169.85 to 90.60 mg/100 g. This was because of thermal degradation and leaching of phenolics into the endosperm, forming macromolecule complexes like protein, which limits their extractability. The study [110] evaluating the effect of germination (30 °C for 48 h) and roasting on TPC in white finger millets found that pan-roasted flour (120 °C for 5 min) contains the highest TPC and TFC of 0.11 and 0.84 mg QE/100 g, respectively. Germination reduced TPC and TFC (0.02 and 0.641 mg QE/100 g d.m), respectively, due to the hydrolysing effect on phenolic compounds, whereas roasting enhanced them through the Maillard reaction. The increase in TPC and TFC due to roasting has been duly noted in a study [130] on germinated quinoa seeds, where TPC 2.13 mg/100 g dw and TFC 0.37 mg/100 g dw in raw quinoa seeds increased to 39.23 mg/100 g dw and 4.10 mg/100 g dw on roasting germinated quinoa at a temperature of 145 °C. The antioxidant properties of advanced and intermediate Maillard reaction products (MRPs) were also supported by a study [131], which found that the TPC of cashew nuts, kernels, and testa enhanced during roasting. While TPC increased in both soluble and bound extracts, degradation was observed in proanthocyanidins (condensed tannin).

Roasting at higher temperatures increases antioxidant capacity, while prolonged roasting decreases the overall antioxidant content in processed millets [93]. Generally, roasting enhances the solubility of phenolic compounds in plant food, thereby improving their antioxidant activity [107]. However, phenolic acids in millets are susceptible to oxidation at high temperatures exceeding 110 °C [103]. For example, roasted finger millet showed a substantial reduction in phenolic content from 314.2 (unroasted millet) to 223.3 mg/100 g; d.w, and antioxidant activity from 89.8 to 86.7% [99]. Similarly, roasting barnyard millets at 70–80 °C for 6–7 min decreased phenolic compounds from 1.36 to 1.02 mg/100 g, indicating thermal degradation and evaporation of phenolics. In another study, barnyard millets showed a reduction in TPC from 1.59 g/mg; d.w (raw) to 0.31, 0.68, and 0.25 g/mg; d.w and TFC from 3.51 to 1.21, 0.68, and 0.16 mg/g; d.w after dehulling, roasting, and boiling, respectively. The DPPH and FRAP activities were lower in dehulled (0.07, 0.78), roasted (0.25, 0.85), and boiled samples (0.21, 0.28) compared to raw millet (0.28, 3.08 mg AAE/g DW) due to thermal degradation of phenolic compounds [105]. The positive effect of thermal treatments on TPC was reported in another study [103], where broom millet was roasted (110 °C for 10 min after soaking for 6 h), steamed (110 °C for 10 min), puffed (110 °C at 1 MPa), and extruded (feed rate (30 g/min, 150 pm, 110 °C with 80–100 bar pressure)). The total phenols (670 mg/100 g on dry basis) and total flavonoids (391 mg/100 g on dry basis) were highest in roasted samples, followed by puffed (TPC 645 mg/100 g; d.w, TFC 304 mg/100 g; d.w), extruded (TPC 455 mg/100 g; d.w, TFC 219 mg/100 g; d.w), and steamed samples (TPC 315 mg/100 g; d.w, TFC 282 mg/100 g; d.w). Heat treatments induced the hydrolysis of c-glycosyl flavones and released free phenolics. Therefore, thermal processing, including steam, microwave, and roasting, significantly influences the TPC and AOX activity of millets, with varying effects contingent on the type of millet and treatment conditions. Roasting generally increases TPC and antioxidant activity by enhancing the extractability of phenolic compounds through hydrolysis, but excessive heat or prolonged roasting can degrade phenolics and reduce antioxidant properties. Conversely, some methods like boiling or high-temperature microwaving may also decrease the TPC due to phenolic degradation or leaching.

In the process of baking, the effect of heat on phenolics was evaluated [109]. In 100% germinated finger millet flour baked biscuits, the phytic acid decreased from 3.24–2.41 mg/g; d.w, TPC increased from 1.64–1.89 mg GAE/g; d.w, FRAP increased from 4.13–4.17 µM TE/g, and amount of Trolox equivalent decreased from 2.5–2.12 µM TE/g; d.w after baking. These changes suggest that baking enhances phenolic content and AOX activity in millet-based products because of the thermal breakdown of cellular structures and the release of extractable phenolic compounds. In addition, antinutrients such as phytic acid are degraded or inactivated by heat, thus improving the overall nutrient profile and health benefits of baked products.

The extrusion process in millets is found to increase the TPC and AOX. For example, a study [111] investigated extrusion at 110 °C with 350 rpm to develop composite bread from finger millet flour. The highest TPC of 80.7 mg GAE/100 g and antioxidant activity (AOX) of 10.7 mM TE/100 g dw, compared to the control bread (TPC of 20 mg FE/100 g and AOX of 9 mM TE/100 g dw), was recorded. Additionally, the impact of barrel temperature (ranging from 90–150 °C), feed moisture content (between 17–25%), and screw speed (from 170–250 rpm) was assessed [112]. The AOX activity increased at higher screw speeds but decreased with increasing moisture content and temperature. In extruded millet, antioxidant activity ranged from 16.5 to 31.4 mM/g and was significantly affected by temperature. The increase in TPC and AOX with extrusion was because of the breakdown of cellular structures and the release of bound phenolic compounds. Higher screw speeds enhance the phenolic release, while increased moisture content and temperature may degrade the phenolics. Therefore, optimizing extrusion conditions could significantly enhance the nutritional profile of millet-based products. These studies demonstrated that thermal processing methods, such as parboiling, roasting, pressure cooking, and extrusion, significantly influence the phenolic content and AOX activity in millets. Thermal treatments promote the hydrolysis of phenolic compounds, enhancing their bioavailability and antioxidant properties. Most methods reduce antinutritional factors, making millet-based foods more nutritious and beneficial for health.

#### 4.1.3. Effect of Non-Thermal Processing on Polyphenols

The effect of non-thermal processing, such as irradiation, on phenolics in finger millet slurry was investigated at various doses (2.5, 5, 7.5, and 10 kGy from a cobalt-60 source at a dosage rate of 2.5 kGy/h) of γ-radiation. The treatment reduced the phenolic content, with CO-14 and CO-15 varieties showing reductions of 23.71% and 29.39%, respectively, at 10 kGy compared to untreated samples. Similarly, the TFC of CO-14 and CO-15 finger millet flour increased by 24.82% and 19.25%, respectively, at a higher dosage of 10 kGy, though the increase was insignificant [114]. Similarly, a study [115] reported significant reductions in polyphenol content in sorghum grains after gamma and electron beam irradiation, which was attributed to increased protein complex formation. Interestingly, infrared (IR) exposure for 120 s increased polyphenols from 1155 to 1228 mg CE/100 g, possibly due to the thermal alteration of cell components or decomposition of insoluble phenolic compounds. Additionally, γ-ray irradiation improved millet’s antioxidant properties by enhancing antioxidant enzymes [4]. In another method involving cold plasma, the TPC of 398.1 mg GAE/100 g of dry matter and TFC of 278.6 mg QE/100 g of dry matter, along with AOA of 7.61% in control pearl millet flour, decreased with increasing power levels and treatment durations, applied at different power levels (20–30 kV) and durations (10–20 min). The cold plasma generated ROS (Reactive Oxygen Species) and ozone that degrade aromatic rings, resulting in the loss of phenols, flavonoids, and hence antioxidant activity [118]. Similarly, the treatment with 30 kV for 30 min led to a notable increase in phenol content from 229.33 to 280.54 mg GA per 100 g. Additionally, the flavonoid content increased from 173.75 to 244.71 mg QE per 100 g; d.w in a study on barnyard millet flour using multipin atmospheric cold plasma (MACP) treatment [121]. Increasing the voltage and durations enhanced the breakage of covalent bonds with larger molecules, releasing phenols and flavonoids. Additionally, these conditions disrupted the cell membranes, thereby increasing the extractability of bioactive compounds. In the case of ozonation of red sorghum flour at various doses (32.0, 38.0, 44.0, and 50.0 g/kg), the TPC increased from 226.34 to 276.23 mg GAE/100 g; d.w, and FRAP activity enhanced from 8.14 to 12.65 µmol TE/g; d.w. However, ozonation reduced the TFC from 605.26 to 582.15 mg QE/100 g and decreased DPPH activity from 10.90 to 9.61% RSA. This was attributed to ozone-induced oxidation, which reduces the flavonoid levels and inhibits peroxidase and PPO enzyme activities, thereby preventing the loss of phenols [117].

In ultrasound application, the detrimental effect on total phenols and major phenolic constituents at higher ultrasound frequency and duration was recorded [89]. In this study, sorghum was sonicated at various amplitudes (40, 60%) and duration (5, 10 min). The treatment at 40% for 5 min increased TPC from 1.18 to 1.26 mg GAE/g of dry matter, DPPH radical scavenging activity from 83.7% to 89.1%, and FRAP values from 0.029 to 0.031 mM FE/g of dry matter. However, these values declined with a further increase in amplitude (60%) and duration (10 min). The germination (%) results followed a similar trend, with the highest germination (94%) obtained for 40% for 5 min, and decreased linearly for prolonged treatments at higher amplitude. These findings underscore the potential of non-thermal methods to modulate phenolic content and antioxidant properties in millets, suggesting that optimizing treatment parameters could maximize health benefits while minimizing losses in phenolic compounds.

#### 4.1.4. Combination of Techniques and Their Effect on Polyphenols

The application of high-pressure assisted soaking in foxtail millets has demonstrated the enhancement of hydration kinetics and reduction of antinutrients and allergens. The combination of temperature (20, 40, 60, 80 °C) and pressure (200, 400, and 600 MPa) treatments for 30, 60, 90, and 120 min was evaluated. The highest TPC of 3.21 mg GAE/g on dry basis was recorded at 600 MPa for 120 min treatment at 20 °C. This was achieved through prolonged pressure and time while minimizing temperature to prevent oxidation of free polyphenols. Higher temperatures oxidize the leached free polyphenols while increasing pressure and time overcomes the cell walls and hydrophobic bonds to improve the extraction of polyphenols [59]. In another study, finger millet was subjected to ultrasound-assisted hydration at variable amplitude (30, 50, 70%), treatment time (10, 20, 30 min), and grain-to-water ratio (1:3, 1:4.5, 1:6). The study demonstrated a significant reduction in phytates from 486.2 to 185 mg/100 g. The combination of high ultrasound amplitude, prolonged hydration time, and a low grain-to-water ratio substantially decreased TPC due to the cavitation effects effectively disrupting the cell wall and causing phenols to leach into the water. The lowest TPC of 190.9 mg GAE/100 on dry basis and highest 308.8 mg GAE/100 on dry basis were recorded at 70% for 20 min and 30% for 10 min treatment, respectively. In ultrasound-assisted hydration, the TPC was higher (255 mg TAE/100 g on db) compared to conventional hydration with a TPC of 253.30 mg TAE/100 g on db. The conversion of tannic acid to gallic acid and lower leaching of condensed tannins during sonication was the probable reason for these results [74]. Similarly, the enzyme treatment followed by ultrasonication, as opposed to ultrasonication alone, was studied for polyphenol extraction [63]. Xylanase (50,000 U/mL) and cellulase (187,500 U/mL) were added at different concentrations and incubated at 50 °C for 2 h. Pretreatment with xylanase yielded 20% more phenols (47.9 g/100 g; d.w of finger millet seed coat (FMSC) compared to cellulase enzyme (37.7 g/100 g; d.w of finger millet seed coat). This was attributed to the higher incidence of xylan-containing polysaccharides in finger millets, often bound to ferulic acids. The synergistic effect between enzyme pretreatment and ultrasonication substantially increased the release and availability of bound and unbound phenolics, including cyanidin 3-malonylglucoside, luteolin, catechin, etc. Ultrasonication-assisted enzyme treatment proved superior due to the unique phenolic composition, including 5-O-caffeoyl shikimic acid and dicaffeoyl shikimic acid that were undetected in heat reflux or conventional ultrasound treatments [63]. In conclusion, the integration of novel processing methods such as high-pressure-, ultrasound-, and enzymatic-assisted treatments are promising methods for reducing antinutrients in millets while enhancing the extraction of beneficial phenolic compounds. Further research into optimizing the combination techniques would enhance the nutritional benefits, promoting the development of functional foods and dietary strategies for improved health benefits.

### 4.2. Effect of Processing on Tannins

Tannins are considered antinutrients because these compounds chelate with minerals and inhibit protein digestion [132]. Tannins or proanthocyanidins interact with enzymes and decrease the bioavailability and nutritional value of proteins and minerals. They also inhibit the activity of digestive enzymes, leading to decreased protein digestibility. Additionally, tannins chelate with minerals like calcium, interfering with their absorption and subsequently reducing their bioavailability. In contrast, tannins have proven to be useful in cancer therapy applications, reduction of neuronal deaths in Alzheimer’s, and prevention of renal calculi [133,134,135]. Tannin concentration could be reduced by processing interventions before the consumption of foods containing excessive tannins.

#### 4.2.1. Effect of Physiochemical and Bioprocessing on Tannins

Bioprocessing methods such as germination, sprouting, and soaking are effective tannin reduction methods in various millet and sorghum grains. For instance, a study [35] reported a tannin reduction in finger millet (2 to 0.55 mg/g), foxtail (1.9 to 1.2 mg/g), pearl (1.8 to 0.53 mg/g), kodo (1.4 to 0.52 mg/g), and little millet (1.2 to 0.34 mg/g). Similarly, increasing sprouting time from 0 to 48 h decreased the condensed tannins from 3 to 1.1% in finger millet, 1.5 to 0.67% in foxtail millet, 3.7 to 1.2% in kodo millet, 1.8 to 0.6% in little millet, and 1.5 to 0.7% in proso millet. Additionally, soaking finger millet at temperatures ranging from 30–50 °C for 0 to 24 h reduced the antinutrients by 40 to 50% across all temperatures and durations [79]. Furthermore, in untreated foxtail millets, the tannin levels (86.5 mg/100 g) were reduced by soaking (73.3 mg/100 g for 12 h), germinating (53.5 mg/100 g for 12 h), and fermenting (60.3 mg/100 g for 20 h) at 38 °C [26]. In kodo millet, tannins decreased from 1.603 to 0.234 mg/100 g after 13 h of germination, primarily due to enhanced activity of phytase during germination [93]. Sorghum grains with tannin content (0.12–4.4 mg/g) showed a reduction by 13.2–50% after steeping (6 h) and sprouting (32 °C for 0, 48, and 96 h). Tannins were further reduced by 26.3% after 96 h of germination and malting, eventually becoming undetectable [62]. In foxtail and proso millet, tannins significantly dropped from 0.33 to 0.02 mg/g. This reduction was observed after 15 h of soaking and germination at 24 °C for 4 days. These reductions were attributed to the conversion of tannins into precursors for the biosynthesis of various phenolic acids and flavan-3-ols [84]. In a parallel study, finger and pearl millet subjected to germination (48 h) at 35 °C indicated a substantial reduction in tannin content. The germinated pearl and finger millet flour reduced tannins from 0.61 mg/g to 0.45 mg/g and 1.61 mg/g to 1.04 mg/g, respectively. The tannin reduction was attributed to the leaching of polyphenols into the soaking water and elevated enzymatic activity during germination [50]. Germinated millet flour (360.5 mg/100 g) and popped millet flour (610 mg/100 g) showed lower tannin content compared to whole millet flour (870.9 mg/100 g), enhancing acceptability, digestibility, and bioavailability [87].

Finger millet was soaked overnight and germinated at 25 °C for 12 to 96 h. After 48 h germination tannins reduced from 173.7 to 64.3 mg/100g; d.w and later increased to 94.6 mg/100g; d.w after 96 h of germination. It was interesting to note that prolonged germination beyond 72 h led to high malting losses and increased antinutrients, which must be avoided [41]. Contrary to the findings, the tannin content of whole barnyard (4.72%), foxtail millet (4.9%), and little millet (6.1%) increased to 5.78, 6.2, and 9.1%, respectively, in germinated flour after germinating at 35 °C and 30 °C for 24 h. It was reported that the tannins are primarily present in the seed coats and are less affected by germination [5]. A study analyzed the effect of soaking, steaming, pearling, and size reduction in pearl millet on antinutritional factors. The results indicated a significant reduction of tannin levels by 7.9% during these processes. Pearling reduced tannins by 29.92%, again suggesting that tannins are concentrated in the outer layers of millets. The high tannin levels in the outer layer contribute to the bitterness of whole pearl millet flour [136]. In this regard, decortication effectively reduced tannins from 343.91 to 174.4 mg/100 g; d.w due to the loss of outer layers [57]; this method also improves the edibility of grains. The impact of supplementing millet flour with moringa seed flour (MSF) and cooking on the tannin content was examined [137]. This study revealed significant differences in tannin concentration between raw and composite flours. The variation in tannin levels was because of the replacement of millet flour with high tannins with moringa seed flour, suggesting an alternate way to reduce excessive tannins in the final product.

The solid-state fermentation (SSF) of pearl millets for 10 days with *Rhizopus azygosporus* exhibited to a substantial increase in condensed tannins from 101.3 to 176.7 mg CE/100 g. During fermentation, the concentration of bioactive compounds, particularly condensed tannins, is significantly enhanced [56]. Similarly, in germination and fermentation processes, the tannin content in raw millet (0.5 g/100 g; d.w) increased equally in both germinated and sprouted samples to 0.68 g/100 g; d.w. This enhancement suggests that fermentation may facilitate the polymerization of catechins into tannins [70]. On the contrary, fermentation of sorghum flour by three LAB strains (*L. brevis*, *L. bulgaricus*, *L. casei*) with and without NaOH immersion showed that combined fermentation with NaOH immersion treatment offers the most significant reduction in tannin content, from 6.16% to 0.063% [67]. Similarly, in fermentation with *L. brevis* for 12 to 36 h, tannin content in the foxtail millet decreased from 0.48 to a range of 0.28–0.23 mg/g [48]. A study on pearl and finger millets subjected to various treatments, including soaking (ST) at 28 °C, germination (GT) at 25 °C for 48–72 h, microwave treatment (MT) at 900 W for 40–100 s, open fermentation (OF) for 48 h, and closed fermentation (CF), involving inoculation with *Aspergillus niger* and fermentation at 29 °C for 48 h. The tannin and phytic acid were substantially reduced compared to untreated (UT) millets. The least reduction in tannin content (38–45%) was observed in millets treated with MT and soaking, while the highest reduction (95–96.5%) was in OF or CF with almost a 90% reduction in tannin content for both millet varieties [76].

Proso millets underwent processes including soaking for 12 h, germination for 48 h, fermentation for 20 h, and a combined treatment of three methods. The combined treatment effectively reduced tannins from 73.4 to 26.5 mg TA/100 g of dry flour; d.w. Additionally, the leaching of phytates and tannins into the water was facilitated during soaking [26]. Similarly, tannin content decreased by 20 and 38% in pearl millet and by 13 and 37% in finger millet following fermentation and germination, respectively [80]. During the initial 24 h of fermentation, there was a rapid reduction of 34%, and after complete fermentation of 72 h, the reduction amplified to 41%. Germination led to a 52% decrease in tannin levels in sorghum [83]. It was evident that bioprocessing methods offer promising strategies to selectively remove tannins from outer layers or reduce tannin concentration, enhancing the nutritional value of foods. These methods also improve digestibility and bioavailability and increase the overall acceptability and health benefits of millets through processing. Integrating these techniques into food processing practices could significantly optimize the nutritional quality of staple foods.

#### 4.2.2. Effect of Thermal Processing on Tannins

The effect of steaming on phenolics, flavonoids, and tannins in little millet flour was investigated. The initial tannin content, ranging from 283.4 to 308.6 mg 100 g^−1^ (d.w), increased to 283.4–336.8 mg 100 g^−1^ (d.w) after the dry heat process through roasting [138]. In two studies, pearl millet varieties subjected to 72 h of malting and 48 h of sprouting resulted in tannin reduction. However, traditionally fermented pearl millet flour-based lahoh enhanced the tannin content, which negatively impacted the loaf’s nutritional profile. On the other hand, dry heat treatment of whole pearl millet reduced the tannin content from 1.52 to 1.30 g/100 g; d.w due to thermal degradation [113,139]. Pearl millet processed using various methods exhibited a notable decrease in tannin content by 38% for pressure cooking, 55% for open pan boiling for 5 min, and 31% and 36% for microwave heating at 450 W and 600 W, respectively [129]. Additionally, roasting barnyard millets at 70–80 °C for 6–7 min significantly improved their nutritional composition, reducing tannin levels from 1.36 to 1.02 mg/100 g. The reduction in nutrients was mainly due to thermal degradation and the evaporation of these compounds during roasting to a small extent [51]. The raw and composite millet flours supplemented with 5, 10, and 15% moringa seed flour (MSF) resulted in reduced tannin content to 18, 7, 8, and 9 mg/100 g, respectively [140]. Roasting reduces antinutrients such as tannins in foxtail millet from 221.1 to 92.4 mg CAE/100 g of dry matter, particularly during the milling of roasted samples. Popping, a high-temperature short-time (HTST) processing technique used to prepare expanded cereals for snacking, breakfast, and ready-to-eat foods, decreased the tannins from 870.8 to 610.2 mg/100 g [93]. Thermal treatments such as roasting, pressure cooking, and popping effectively reduce tannin content in millets, enhancing their nutritional quality. These processes lower antinutrient levels, with roasting and popping show significant reductions, thereby enhancing the health benefits and functional properties of millet-based foods.

#### 4.2.3. Effect of Non-Thermal Processing on Tannins

Among non-thermal techniques, the application of cold plasma and irradiation to reduce antinutrients has been investigated. The atmospheric cold plasma treatment on pearl millet flours demonstrated a maximum tannin reduction from 0.98 (control) to 0.81 g tannin acid/100 g at a power level of 30 kV for 20 min [118]. The cold plasma treatment demonstrated the potential to modify the functional groups that constitute tannins, and the depletion of tannins is likely due to the oxidation of reactive species that cleave glycosidic in tannins. Another study [115] evaluated the impact of infrared (IR), gamma, and electron beam irradiation on tannins in sorghum grain. The sorghum grain subjected to IR treatment at 1000 W IR lamp for 60 s resulted in a substantial reduction of tannin levels from 921 to 393 mg/g; d.w. Similarly, gamma and electron beam radiation offered the highest tannin reduction by 79% and 73%, respectively, at the highest dosage of 30 kGy. Similarly, another study confirmed the tannin reduction at a higher radiation dosage [114]. In this study, the tannin content of 249.71 mg/100 g; d.w (CO-14) and 256.72 mg/100 g; d.w (CO-15) in the control samples decreased to 215.87 and 215.35 at 5 kGy, and further to 192.98 and 192.06 mg/100 g; d.w at 10 kGy, progressively with increasing dose. The tannin reduction in irradiation treatments is attributed to the generation of hydroxyl and superoxide anions, which leads to protein degradation, changes in chemical reactivity, and a significant increase in tannin solubility. While fewer studies explain the action of the electron beam in tannin reduction, it is surmised that degradation, polymerization, and modification of tannin structure in a way that inhibits the ability to bind with proteins as a result of ionization, excitation of molecules, and free radical formation are probable mechanisms [141,142]. The tannic acid content of kodo millet was observed to decrease significantly with increasing germination time (12–72 h), ultrasonication time (10–30 min), and temperature (20–40 °C). Optimal ultrasonication for 30 min with 72 h for germination at 40 °C preserves the antioxidant properties, and tannin content decreased from 4.94 to 2.74 mg TAE/100 g. The ultrasonication process enhances the interaction between tannins and hydroxyl radicals, leading to the conversion of tannic acid into gallic acid [34]. Non-thermal techniques demonstrate potential means to reduce tannin content in millets. Cold plasma works through oxidative reactive species that cleave glycosidic bonds in tannins, while irradiation treatments reduce tannins by generating reactive species that increase tannin solubility and degrade their structure. Ultrasonication followed by germination also decreases tannins by promoting interaction with radicals and converting tannic acid into gallic acid.

#### 4.2.4. Combination Techniques and Their Impact on Tannins

High pressure ruptures cellular microstructures, eventually facilitating the leaching or extraction of cellular compounds. The impact of high pressure on antinutrients suggests that High Pressure processing (HPP) is a promising method for reducing antinutrients in millets [143]. For example, both germinated and non-germinated foxtail millet samples were subjected to high-pressure treatments at 200, 400, and 600 MPa at temperatures of 20, 40, 60, and 80 °C for 30, 60, 90, and 120 min. The tannin content was reduced by 45% due to the high pressure exerted on the cells, which eventually expels the tannins to the extracellular medium [59]. Finger millet was subjected to ultrasound-assisted hydration treatment, including variable amplitude (30–70%), duration (10–20 min), and grain-water ratio (1:3 to 1:6). The ultrasound treatment at 66% intensity for 26 min with a grain-water ratio of 1:3 resulted in the highest reduction in tannin content by 62.8%. The study demonstrated that increase in water content significantly reduced tannin levels from 187 to 35 mg TAE/100 g on dry basis. The reduction in tannins was attributed to the conversion of hydrolysable tannic acid into gallic acid and the leaching of condensed tannins [74]. The synergic effect of hydrothermal and milling processes on finger millet (soaked for 10 h at 30 °C, followed by steaming at 98 kPa for 20 min) was investigated by [106]. Subsequently, milling was conducted, varying the roller speed (ranging from 1000 to 1400 rpm), residence time (10 to 14 min), and dispersed density (430 to 560 kg/m^3^). Significant reductions in tannin content (10.3 to 50%) were observed for combined pearling and hydrothermal treatment. Notably, an increase in residence time (from 12 to 14 min) resulted in the maximum reduction in tannins (ranging from 2.5 to 3.5 ppm). Under the optimized milling conditions with roller speed of 1001.3 rpm, 13.9 min residence time, 560 kg/m^3^ dispersed density, and 7.4% degree of pearling, the tannin content notably decreased to 3.19 ppm, compared to unprocessed millet, which contained 4.14 ppm. This reduction in tannins was mainly because of the separation of the bran layer during pearling, indicating the accumulation of tannins in the outer layers of the millet. The combination techniques employed to reduce antinutrients, such as the integration of high-pressure treatments, ultrasound-assisted hydration, and hydrothermal treatment with milling, have shown significant effectiveness in reducing tannin content compared to single techniques. These methods leverage synergistic effects, leading to more substantial reductions in antinutrients. The implications of these outcomes indicate that a combination of processing methods significantly reduces tannin content, improving the nutritional quality of millets. This approach offers promising strategies for improving food products by retaining beneficial compounds and reducing undesirable ones.

### 4.3. Effect of Processing on Phytates/Phytic Acid

The phytic acid (PA) is highly reactive due to its negative charge nature over a broad range of pH. These compounds quickly react with opposite charge ions like minerals, forming insoluble complexes that are less accessible for absorption or digestion in the human body [144]. Due to this behavior of phytates, it is recognized as an antinutrient. Myoinositol hexaphosphate is the most prevalent form of phytate found in plants, a way in which the seeds store phosphorus in protein vacuoles of the aleurone layer for future Adenosine triphosphate (ATP) synthesis [145]. While phytic acid decreases the solubility and bioavailability of nutrients, it also supports cholesterol metabolism and helps regulate cholesterol levels [36]. Millet varieties such as finger, foxtail, pearl, and proso are rich in phytates. During processing and digestion, myoinositol hexaphosphate, the predominant form of phytate, is degraded into penta-, tetra-, and triphosphates, which exhibit poor chelation ability with minerals.

#### 4.3.1. Effect of Physiochemical and Bioprocessing Methods on Phytates

It is generally noticed that phytates are concentrated in the outer layer of millets. Due to this reason, a study [126] stated that refined flour from debranned pearl millet contained substantially low phytates, proving the accumulation of phytates in the outer layers. Additionally, a study reported reduced phytic acid in chapati (Indian bread) made from milled millet flour [93]. Another processing method, soaking, activates the phytase enzyme, leading to a substantial reduction in phytic acid. Soaking at temperatures between 45 and 65 °C and pH of 5 and 6 effectively hydrolyzed the phytates, thereby enhancing the bioavailability of minerals [132]. The phytic acid was decreased from 7.8 to 4.8 mg/g on dry basis (finger millet), 7.2 to 4.1 mg/g on dry basis (foxtail millet), 8.3 to 4.1 mg/g on dry basis (kodo millet), 8.3 to 4.5 mg/g on dry basis (little millet), and 9.3 to 5.2 mg/g on dry basis (proso millet) upon sprouting [52]. Similarly, after 13 h of germination in kodo millet, the phytic acid decreased from 1.344 to 0.997 mol/kg; d.w. This reduction was attributed to increased phytase activity during germination [93]. In another study, finger millets were soaked for 12 h at 30 °C, then germinated for 12, 24, and 36 h at 30 °C, 34 °C, and 37 °C with 80% relative humidity. Phytic acid (mg/100 g; d.w) in raw finger millet (676.7 mg/100 g; d.w) was reduced to 587 mg/100 g; d.w after 12 h of soaking and further decreased to 238.4 mg/100 g; d.w after 36 h of germination at 37 °C [58]. Phytate content (459–1097 mg/100 g; d.w) was reduced by 32.7–56.3% following the germination and malting of sorghum grains [62]. Germination is a promising method to reduce phytic acid levels. A few studies [40,86] on germination showed that the phytate content in finger millet decreased from 51.67 to 35.00 mg/100 g after 48 h, and further to 21.67 mg/100 g at 96 h, and in foxtail millet it dropped from 0.341 to 0.102 mol/kg. Similarly, foxtail millet germinated at various temperatures and durations with soaking times between 5.27 and 18.73 h exhibited a reduction in phytate content from 0.34 to 0.1 mol/kg [86]. Foxtail and proso millet were soaked for 15 h, then germinated at 24 °C for 4 days. In finger millet and proso millet, phytic acid content notably decreased from 6.4 to 2.6 mg/g; d.w and 8.09 to 4.8 mg/g; d.w post-germination [84]. In germinated finger millet flour and pearl millet flour, the phytate content decreased from 15.98 to 9.77 mg/g and 15.11 mg/g to 6.34 mg/g, respectively. The decrease in phytate content was due to the hydrolysis of phytate phosphorus into inositol monophosphate. This process was facilitated by enhanced phytase activity during germination and further aided by leaching during the hydration phase [50].

Various processing methods were applied to white finger millet varieties; soaking (12, 24, and 48 h), malting (overnight steeping, germination at 25 °C for 12, 24, and 48 h, drying at 60 °C for 6 h), and popping (9% moisture content, 170–200 °C). Results showed that soaking for 0–48 h reduced initial phytate content (266 mg/100 g) by 16.9%, while malting for 12 h and 28 h linearly reduced phytate by 11.7% and 50.6%, respectively. Popping reduced phytate by 17.1% from the initial value of 266 mg/100 g. The highest average reduction of phytate was recorded for 48 h malting (266–132 mg/100 g), followed by popping (266–221 mg/100 g) and 48 h soaking (266–221 mg/100 g) for different varieties. Longer intervals of soaking, malting, and popping were observed to enhance nutrient availability by inactivating antinutrients [60]. Similarly, after 48 h germination followed by popping, both the germinated (238.5 mg/100 g) and popped (333.1 mg/100 g) millet flour showed lower phytic acid content compared to whole millet flour (851.4 mg/100 g) [87]. Phytic acid in the untreated sample (7.4 mg/100 g) was reduced by soaking (6.3 mg/100 g), germinating (4.5 mg/100 g), and fermenting (5.2 mg/100 g) [26]. The two pearl millet varieties (Kalukombu and Rabi Bajra) including phytate contents of 0.78 and 0.57 g/100 g, respectively, reduced by more than 50% after germination in both varieties [126]. In both pearl and finger millets, various treatments were applied, including soaking (ST), germination (GT), microwave treatment (MT), open fermentation (OF), and closed fermentation (CF). Among these treatments, closed fermentation (CF) consistently exhibited the highest reduction in phytic acids (ranging 76–86%), while soaking or microwave treatment (MT) resulted in the least reduction (ranging 26–27%) in both millets [76].

In another processing method, the fermentation of millets creates an optimum pH (<4.5) conducive to the degradation of phytic acid. A reduction in phytic acid content of 17.8–18.9% was observed in pearl millet rabadi when fermented for 12 and 24 h across all soaking periods with increased temperature from 30 to 45 °C. Specifically, natural fermentation at higher temperatures (45 °C) led to a significant reduction in phytate compared to fermentation at lower temperatures (37.5 and 30 °C) [69]. The finger millets were fermented with LAB, yeast, and combination treatments, both with and without (NH4)_2_SO_4_. The phytic acid levels were reduced by 20.8–67% across all treatments, with the highest reduction observed in the *L. brevis* treatment. This reduction can be attributed to increased phytase activity and the hydrolysis of polyphenolic compounds by polyphenol oxidase [6]. Similarly, during fermentation with *L. brevis* for 12 to 36 h, phytic acid content in foxtail millet decreased from 325 to 314 mg/100 g [48]. Similarly, the phytate level was reduced by 88.3% when pearl millet was germinated and fermented with mixed pure cultures of *S. diasticus*, *S. cerevisiae*, *L. brevis*, and *L. fermentum* at 30 °C for 72 h. Fermentation of sorghum flour by three LAB strains (*L. brevis*, *L. bulgaricus*, *L. casei*) with and without NaOH immersion, as well as combined treatment, resulted in significant reductions in phytic acid from 11.9% to 0.11%. Interestingly, there was no significant difference observed in phytic acid reduction among different LAB species [67]. These research outcomes highlight the potential of fermentation in addition to other effective methods to reduce phytic acid. Germination and fermentation additionally enhance the nutritional value of millet through chemical changes and mitigating the antinutritional factors [132].

In the process involving steeping (28 °C for 16 h), fermentation (72 h), germination (12 h), and combination treatment, both pearl and finger millet noted phytic acid reduction. The reduction results were 42% and 24% for steeping, 54% and 34% for fermentation, and 70% and 75% for germination for pearl millet and finger millet, respectively [80]. Similarly, proso millets processed by all three methods resulted in a reduction of phytic acid content ranging from 8.77 to 2.4 mg/g across all treatments [26]. Likewise, phytic acids, during the initial 24 h of fermentation, reported a reduction of 23%, and after complete fermentation of 72 h, further reduced to 38%. Steeping reduced phytic acid by 21%, while germination resulted in a more substantial reduction of 46% [83]. The above studies indicate that combining biological methods like soaking, germination, and fermentation offers more efficient combination techniques to mitigate phytic acid levels in millets than a single technique. These combined approaches not only enhance nutrient bioavailability but also lead to substantial chemical changes that remove antinutritional factors. The synergy of multiple methods maximizes phytase activity and hydrolysis processes, significantly improving the nutritional quality of millets. This multi-step approach holds great promise for developing functional millet-based foods with enhanced health benefits and better mineral absorption, addressing both nutritional and antinutritional challenges.

#### 4.3.2. Effect of Thermal Processing on Phytates

Combining heating and soaking techniques has proven more effective in reducing phytic acid in millets than soaking alone. For instance, roasting foxtail millet has been shown to reduce the phytic acid in foxtail millet from 306 to 180.5 mg/100 g; d.w. Soaking of pearl millet for 9 and 12 h resulted in a significant decline in phytic acid from 683.07 to 624.44 (9 h) and further to 616.72 mg/100 g dry weight basis; d.w (12 h), and this was attributed to the increase in soaking time. Phytic acid (683.07 mg/100 g; d.w) was seen to be reduced significantly after both 5 min (608.51 mg/100 g; d.w) and 10 min (603.87 mg/100 g; d.w) of pressure cooking. It was also reported that after steaming for 5 and 10 min, the phytic acid decreased considerably from 683.07 to 625.34 mg/100 g; d.w and 616.9 mg/100 g; d.w, respectively. The reductions were attributed to thermal degradation, leaching, and discarding of soaking and cooking water [93,146]. Popping, which is a high-temperature short time (HTST) processing method used to obtain RTE food products, has resulted in the reduction of phytic acid from 851.4 to 333.1 mg/100 g [93]. Another study, ref. [144], revealed that traditional cooking methods may not effectively reduce phytates in pearl millet due to the heat stability of phytate. No substantial phytate reduction is expected during cooking or any other traditional heat processing procedure unless the cooking water is discarded. Heat inactivates endogenous phytases during cooking, thereby preventing the breakdown of phytate. Effective reduction typically requires high-temperature processing, which may not be achievable in typical cooking methods.

#### 4.3.3. Effect of Non-Thermal Processing on Phytates

As discussed earlier, a study [115] highlighted the significant decrease in phytates in sorghum grains following irradiation with IR, gamma (Co_60_), and electron beam. IR treatment showed a maximum phytic acid loss of 91% at 120 s, while a 90% reduction was achieved with a 30 kGy dose of gamma radiation. These treatments cleave the phytate ring or decompose phytates into inositol and inositol phosphates due to free radical activity, effectively reducing phytates. In another study, cold plasma treatment in foxtail millet at 1 kV and 2 kV for 1, 3, and 5 min accelerated the germination and reduced the phytic acid from 8.89 mg/g to 1.1 mg/g at 2 kV for 3 min. Notably, the same treatment combination resulted in the highest germination percentage of 84.0% [119]. Likewise, cold plasma treatment at 2 kV reduced phytic acid by 57% and 61% in pearl and barnyard millet, respectively [120]. The reduction of phytates by cold plasma is attributed to the loss of the phytate ring by the chemical reactive species formed during cold plasma treatment. Phytates may form insoluble complexes, thereby improving the bioavailability of nutrients.

Additionally, high-pressure soaking of foxtail millets reduced phytate phosphorus and phytic acid on increasing pressure, time, and temperature. Increasing the pressure (400 MPa), temperature (40 °C), and soaking resulted in maximum reduction (67.87%), regardless of soaking duration. The soaking duration did not significantly impact the phytic acid reduction in germinated foxtail millet samples [59]. Another study on the impact of ultrasound (66% intensity, 26 min) and a 1:3 grain-water ratio on hydration rate and antinutrients in finger millet revealed a substantial reduction in phytate content by 66.9%. It is observed that localized heat generation under high-amplitude ultrasound treatment could lead to chemical degradation of phytate [74]. In a similar study, where sorghum grains were subjected to sonication at various amplitudes and times (40% for 5 min, 60% for 5 min, 40% for 10 min, 60% for 10 min) at 35 °C. Phytate levels decreased from 146 to 143.2 mg/g when treated with 40% amplitude sonication for 5 min. However, higher amplitude and longer treatment time resulted in increased phytochemicals [89]. Non-thermal method technologies offer efficient alternatives to traditional processing methods. Irradiation breaks down phytates into inositol and phosphates through free radical activity, while cold plasma reduces phytic acid by cleaving the phytate ring with reactive species. High-pressure soaking and ultrasound also contribute to phytate reduction, with ultrasound inducing chemical degradation due to heat generation. These treatments improve nutrient bioavailability by decreasing phytate levels. Further research into optimizing these technologies for large-scale applications and assessing their long-term effects on food quality and safety is essential for their widespread adoption in food processing.

#### 4.3.4. Effect of Combination Techniques on Phytates

High-pressure-assisted germination reduced phytic acid by 39%. This was due to enhanced phytase enzyme action during germination and high-pressure-assisted leaching of phytic compounds during soaking. The increased solubility of phytic acid complexes during high pressure and temperature treatment further degraded the phytate levels. The optimized treatment conditions included 400 MPa, 60 °C, and a 60-min soaking time [59]. It was noted that the highest phytate reduction of 73.06% was achieved in ultrasound-assisted hydration (USH) of finger millets at 80% amplitude, 20 kHz, with 3:1 water-to-grain ratio, and 20 min. Conversely, USH samples treated at 20% amplitude, 40 kHz, with a water-to-grain ratio of 6:1 for 20 min showed a smaller reduction of 10.30%, indicating the pronounced phytate reduction at high-amplitude treatments. Furthermore, the combined effect of higher amplitude (80%) and longer treatment (30 min) demonstrated the synergistic effect on phytate reduction [147]. The combination of high-pressure- or ultrasound-assisted germination treatments offers promising results in reducing millet phytates. These methods could offer valuable strategies for mitigating antinutritional factors at an industrial scale and improving the bioavailability of essential nutrients.

### 4.4. Effect of Processing on Oxalates

Oxalates, or oxalic acid, form oxalate complexes with sodium, potassium, and calcium, with the latter being insoluble [148]. These insoluble and soluble complexes of oxalates serve as defense mechanisms for plants against grazing by herbivores. Therefore, reducing oxalate levels in millets through certain processing methods holds the potential to enhance mineral bioavailability and their health benefits.

#### 4.4.1. Effect of Physiochemical and Bioprocessing on Oxalates

The oxalate levels in whole pearl millet flour were found to be reduced from 45 to 35 mg/100 g in refined flour, suggesting that the milling process, which involves removing the bran fraction, effectively reduces oxalate levels in the refined product [85]. Similarly, the sprouting of proso millet for 96 h showed a reduction in soluble and total oxalate concentrations and reduced the availability of proteins and minerals during the sprouting [149]. The decrease in oxalic acid during germination has also been reported [93]. Kalukombu and Maharashtra Rabi varieties of bajra millets were examined for oxalate contents, noticing lower (<50 mg/100 g) oxalate content that was further reduced by 24% and 48%, respectively, after germination. The observed decrease in oxalates could be attributed to the leaching of soluble oxalates that occurs during the soaking process [85]. Similarly, foxtail millet (0.56%) and little millet (0.44%), soaked for 12 h and germinated at 35 °C and 30 °C for 24 h, exhibited reduced oxalate content to 0.37% post-germination for both millets. The reduction is associated with the activation of oxalate oxidase during germination, which facilitates conversion of oxalic acid into CO_2_ and H_2_O_2_ [5]. Reducing oxalate levels in millets through bioprocessing, heat treatments, and novel technologies enhances their nutritional value by improving mineral bioavailability, offering potential health benefits of minerals to consumers. Various processing methods like milling, sprouting, and germination play crucial roles in mitigating oxalate content during the primary processing of millets to enhance their bioaccessibility.

#### 4.4.2. Effect of Thermal Processing on Oxalates

The effectiveness of cooking procedures in reducing oxalate content in millets has been investigated [144]. A significant difference in total, soluble, and insoluble oxalate levels was observed between raw and cooked pearl and sorghum millets. In raw and cooked pearl millet, calcium bioavailability and oxalate content were 12.5 mg/100 g; d.w, 20.0 mg/100 g; d.w, 13 mg/100 g; d.w, and 13.4 mg/100 g; d.w, respectively. Boiling water significantly increased oxalate solubility, contributing to the overall reduction during cooking. The oxalates (0.12%) in barnyard millet after processing by dehulling, roasting, and boiling were reduced to 0.105, 0.038, and 0.023%, respectively. The studies demonstrate the potential of thermal processing in degrading oxalates through heat-induced breakdown during various processing stages [105]. For example, germinated flour had 29.8 mg/100 g; dry weight basis of oxalates, and 32.2 mg/100 g; d.w in popped flour compared to a higher oxalate content of 45.8 mg/100 g; d.w in the whole millet flour [87]. Therefore, thermal treatment is an effective method for reducing oxalate levels through heat-induced breakdown and increasing the solubility of oxalates.

#### 4.4.3. Effect of Non-Thermal Methods on Oxalates

There is limited research to understand the effect of non-thermal processing methods on oxalates in millets, except for a study on the application of gamma radiation in finger millet flour. This study demonstrated promising results in reducing oxalate levels and enhancing calcium bioavailability [150]. The irradiated flour exhibited a significant increase in calcium content of 495.8 mg/100 g; d.w compared to non-irradiated flour, containing 418.7 mg/100 g; d.w. The results suggested that gamma radiation effectively degrades oxalates, highlighting the potential of gamma radiation as a non-thermal method for reducing oxalate content and enhancing mineral bioavailability.

### 4.5. Effect of Processing on Saponins

Saponins are a class of glycosidic metabolites characterized by their amphiphilic nature due to the lipophilic aglycone and hydrophilic sugar chain [151]. Saponins have gained research interest due to their diverse applications as stabilizers, foaming agents, and emulsifiers and for exhibiting bioactive properties such as antimicrobial, antioxidant, immunostimulant, and anti-inflammatory properties. However, they also exhibit antinutritional properties by forming complexes with cholesterol and bile salts, reducing the absorption of vitamins A and E. In addition, they bind essential minerals like iron, zinc, and calcium, thus limiting their bioavailability and imparting a bitter taste [152]. Among millet varieties, pearl millet contains the highest concentration of saponins, followed by foxtail, little, finger, and kodo millet [35]. Various processing methods have been employed to effectively reduce saponin content in millets. Saponin breaks down during processing into aglycone, sugar moiety, and pro sapogenins, thereby losing their chemical structure and antinutritional potency.

#### 4.5.1. Effect of Physiochemical and Bioprocessing Methods on Saponins

Several studies have demonstrated that a reduction in saponin content could be achieved by processing, with a substantial reduction recorded for mechanical processes such as milling and polishing. For instance, a study [153] showed that mechanical polishing reduced the saponin content by 50–85% in quinoa, surpassing the results achieved by boiling or steaming. The degree of abrasion during milling, decortication, or polishing of millets influences the amount of saponin reduction. The pericarp contains maximum saponin, and thus polishing facilitates the removal of this layer along the perianth. Despite their functional properties and health benefits, saponins have limited ability to interact, contribute to bitterness, and interfere in their assimilation. The effect of soaking, sprouting, and germination methods on saponins in millets is not consistent with other food grains as per the available literature. The inconsistencies are attributed to the structure of saponins and the proximity to water. Saponins that possess a longer saccharide chain and are in close vicinity to water, such as being concentrated in the seed coats, have a greater tendency to leach out due to lixiviation [154].

Soaking and sprouting of pearl and finger millet lead to a significant enhancement in saponins from 29.117 to 39.530 mg/g; d.w and 24.730 to 34.860 mg/g; d.w, respectively, across 12 h and 24 h treatments [35]. Similarly, malted finger and pearl millet flours showed increased saponin content from 1.80 to 3.19 mg/g; d.w and 0.45 mg/g to 0.86 mg/g; d.w, after germination [155]. The saponin content in pearl and finger millet flour after germination at 35 °C for 48 h increased from 0.55 to 0.96 mg/g and 2.07 to 4.45 mg/g, respectively [50]. In another study [81], the impact of three processing methods, including soaking (12 h), germination (48 h), fermentation (20 h), and a combination of these treatments, on proso millet was investigated. In contrast to previous studies, saponin content decreased significantly from 167 to 29.1 mg/100 g, with subsequent reductions in phytates and tannins. Although soaking mainly reduced saponins, germination and fermentation substantially reduced other antinutritional factors. The study also assessed the impact of three processing methods and their combination at 38 °C in foxtail millet. The results showed a decreased saponin content from 87.3 mg/g of dry flour in the untreated sample to 13.6 mg/g of dry flour and 65.4 mg/g of dry flour for soaking and fermentation, respectively. Interestingly, germination significantly enhanced the saponin content to 113.2 mg/100 g of dry flour. In contrast to other antinutrients, few studies suggest that germination may enhance saponin content, as it promotes endogenous production and stimulates the biosynthesis of enzymes that enhance phytochemical levels.

Soaking and fermentation treatment reduces saponins through leaching and hydrolysis of saponin by β-glucosidase. Similarly, germination studies (15 h soaking, 4 days germination at 24 °C) on foxtail millet and proso millet reported reductions in saponin content from 0.39 to 0.07 mg/g of flour on dry basis and 0.44 to 0.07 mg/g of flour on dry basis, respectively [84]. These reductions were associated with saponins dissolving in water during soaking and the enhanced metabolism of grains during germination. In conclusion, while mechanical processes like polishing and milling effectively reduce saponin content in millets, bioprocessing such as soaking, germination, and fermentation show variable impacts. The inconsistencies are due to the variations in millet varieties, environment, pre-harvest conditions, and analytical methods. Comprehending the principal mechanism of action of the processes employed and the nature of the saponin targeted will assist in optimizing millet processing to enhance nutritional quality.

#### 4.5.2. Effect of Thermal Processing on Saponins

Hot water boiling for 15 min and autoclaving substantially reduced saponin content in quinoa by 64.21% and 13.33%, respectively. In contrast, roasting (6.5 min) and microwave processing (5 min) interestingly enhanced saponin content by 25.15% and 9.31%, respectively. Moist heat was found to be more effective in reducing saponins due to the cleavage of glycosidic bonds and leaching, while dry heat facilitated cell permeation, leading to increased mobilization of saponins [156]. Conversely, another study [99] conducted roasting (10–15 min) and fermentation (24 h) of finger millets (*Eleusine coracana*). Roasting reduced the concentration of saponin, flavonoid, and steroid in millets, while fermentation decreased alkaloid levels. The decrease in saponin content during roasting was attributed to the high temperatures and dry heating during the processes. In sorghum, microwave processing also demonstrated a notable reduction in saponin content from 0.07 to 0.017 mg/100 g of dry matter. In particular, treatment at 700 W for 15 s achieved the highest germination rate (95%) and maximum saponin reduction to 0.007 mg/100 g of dry matter. Microwave processing facilitated the solubilization of saponins and subsequent leaching into water during soaking [104].

Extrusion processing was also noted to reduce the saponin content and other antinutrients such as tannins, phytates, and oxalates due to the high temperature (120 °C), pressure, and shearing force during extrusion. For instance, an extruded breakfast cereal from a combination of finger millet, soybean cake, and carrot pomace in a 60:25:15 ratio (FSC) resulted in the lowest saponin content of 1.03 mg/g compared to 1.33 mg/g in whole finger millet extrudate. However, compositions including finger-millet-soybean cake (75:25) (FS) and finger-millet-carrot pomace (90:10) (FC) increased the saponin levels to 1.80 mg/g and 2.20 mg/g, respectively [157]. From the above studies, it is evident that thermal processing significantly influences the saponin content, and the variations are related to processing conditions.

#### 4.5.3. Effect of Non-Thermal Processing on Saponins

Non-thermal techniques, such as ultrasound, have been extensively used to enhance sprouting in millets and effectively reduce antinutrient levels. For example, ultrasound-assisted sprouting of sorghum under optimal conditions (40% amplitude for 5 min) resulted in the highest germination rate and reduced saponins from 0.15 to 0.09 mg/100 g. This was because of increased intracellular movement of saponins due to ultrasound and subsequent leaching into water [89]. Although non-thermal methods like ultrasound have shown promising results for reducing saponin levels in millets, the research studies reported are limited. However, the optimal ultrasound condition reported in the above study highlights the potential of ultrasound to enhance nutritional quality through innovative non-thermal processing techniques.

### 4.6. Effect of Processing on Other Antinutrients (Lectins, Goitrogen, Haemagglutinins)

Lectins, or haemagglutinins, are proteins that accumulate in plants having an affinity for specific sugar moieties. In millets, they attach reversibly to carbohydrates, resist enzymatic digestion in the GI tract, interfere with nutrient absorption, and cause agglutination [42]. Lectins that can resist degradation by enzymes or heat and agglutinate red blood cells are known as haemagglutinins. Goitrogens, on the other hand, are compounds that suppress thyroid hormone synthesis, interfere with iodine uptake, and can lead to hypothyroidism. Interestingly, the agglutination ability of lectins has been harnessed to target leukemia cells, tumor cells, and neuter pathogens [158,159,160]. In the plant kingdom, goitrogens occur as glucosinolates in plants of the Brassicaceae family and as C-glycosylflavones (C-GFs) in millets [10]. These antinutrients are ubiquitous in millets, which necessitates appropriate processing techniques to reduce or eliminate them and improve palatability.

#### Effect of Physiochemical, Bio, Thermal, and Non-Thermal Processing on Other Antinutrients

Among various bioprocessing methods, pearl millet was soaked and germinated at 30 °C with 90% RH for one day with a 1:3 grain-to-water ratio. The resulting flour enhanced the adipogenesis and thyroid function, indicating the reduction of antithyroids such as C-GFs. The study also observed the inhibition of hypothyroidism and no variation in thyroxine (T4) and triiodothyronine (T3) levels after ingesting germinated flour. It was established that germination reduced goitrogenic polyphenols and enhanced zinc bioavailability, an element essential for the proper functioning of hormone systems [161]. In the case of thermal techniques, there are limited research studies, except for one study on extrudates from finger millet/soybeans processed at 138–141 °C during extrusion, which facilitated in reduction of lectins. Similarly, the application of non-thermal processing to reduce lectins and goitrogens in millets is also scant. However, studies on other agricultural produce highlight fundamental principles and potential applications that can be extended to millets. For instance, the total glycosinolate content of 23.7 mmol/g in canola meal was reduced by 15.6, 30.4, and 49.4% when subjected to γ-irradiation at 10, 20, and 30 kGy, respectively [162,163]. Similarly, in studies on γ-irradiation, phytohemagglutinin in both dry (99.5% dry) and moist red kidney (50%) beans reported a 50% reduction in hemagglutinin and mitogenic activity when irradiated at 30–50 KGy [164]. The reduction of lectins, goitrogens, and haemagglutinins during irradiation is attributed to agglutination, molecular changes, and free radicals that modify their physicochemical properties. The high-pressure processing at 450 MPa was reported to reduce hemagglutinins in red kidney beans by 57.9% due to bond cleavage that reduced agglutination activity [163]. These research insights from other food grains assist in bridging the gap and provide a starting point for developing more targeted studies on millets. The limited study highlights that both bio and thermal methods significantly reduce lectins and goitrogens in millets, improving thyroid function and nutrient bioavailability, whereas non-thermal irradiation and high-pressure processing effectively deactivate these antinutrients through molecular changes and bond cleavage, making millets safer and more nutritious for consumption.

## 5. Nutraceutical Health Benefits of Phytochemical Antinutrients

**Figure 3:**
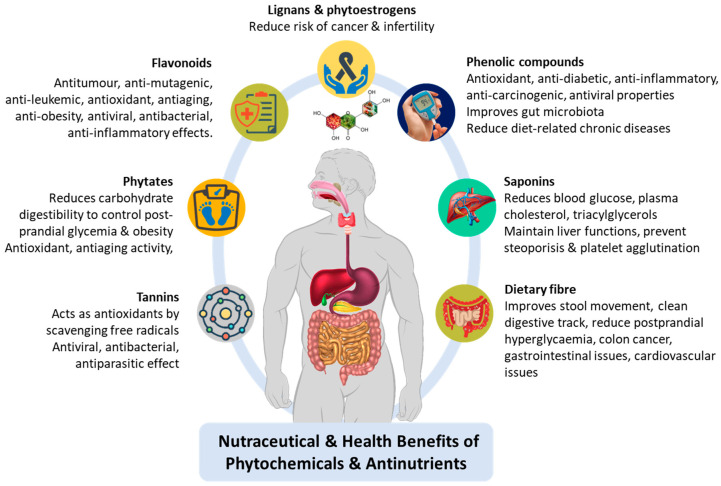
Health benefits of phytochemicals and antinutrients in millets.

Millets are renowned for their nutritional density and are increasingly valued in multigrain and gluten-free cereal products for their substantial health benefits. In addition to their rich nutritional composition, millets contain abundant phytochemicals and bioactive compounds, including polyphenolic compounds, phenolic acids, tannins, and flavonoids. These compounds in millets show significant promise in managing metabolic disorders such as diabetes, cancer, and cardiovascular diseases. Notably, approximately sixty percent of phenolic acids are present as conjugated compounds, while the remainder exists in free or extractable forms [21]. The soluble phenolics found in these millet varieties, particularly flavonoids, have demonstrated potent inhibitory effects on α-glucosidase and α-amylase activities, indicating their potential utility in glycemic control. Polyphenols from millets have been found to prevent neuronal death by downregulating genes associated with Alzheimer’s disease (AD). Additionally, hexane-extracted phenols, saponins, terpenoids, and tannins from pearl millet (Raj 71) inhibited *Pseudomonas aeruginosa* with a zone of inhibition of 15.0 ± 2.5 mm, while the finger millet GPU-45 cultivar demonstrated a maximum zone of inhibition of 18.0 ± 2.5 mm [165,166]. The antinutrients that primarily appear in millets possess pharmacological potential. In addition to being extended as prospective cancer prophylaxis treatments, tannins have proved to be beneficial in alleviating hypertension, preventing the build-up of collagen fibers that leads to myocardial fibrosis and also reducing gingivitis (bleeding of gums) [167,168,169]. These bioactive phytochemicals are primarily concentrated in the outer bran layers of millets alongside essential nutrients like minerals, vitamins, and dietary fibers. Additionally, variations in both phenolic content and antioxidant activities of soluble and bound phenolic extracts of millets were observed, influenced by factors such as variety and cultivated location [170]. Bioactive peptides in millets are generated during the hydrolysis of proteins by enzymes and are recognized for their diverse regulatory functions both in vitro and in vivo. Prolamin peptides from foxtail millet reduced the reactive oxygen species (ROS) as well as malondialdehyde (MDA), exhibiting anti-inflammatory and antioxidant properties [171]. Studies have also shown other multifaceted anti-properties against microbes, oxidation, hypertension, cancer, and diabetes [39]. The dietary fiber content of 15 to 20% in millets comprises non-starchy polysaccharides such as arabinoxylan and β-glucan. Insoluble fibers like cellulose and hemicellulose generally enhance the stool bulk and promote easy bowel movements. On the other hand, soluble fibers, including beta-glucans, arabinoxylans, and pectins, undergo fermentation by gut microflora, producing short-chain fatty acids (SCFAs) [172,173]. SCFAs offer multiple benefits, including a healthy gut, offering energy for colonocytes, and influencing immune function [37]. The health benefits of phytochemicals and antinutrients are summarized in Figure 3.

## 6. Processing Methods Mechanisms of Action, Evaluation, and Recommendation for Optimal Component Balance

In the past decade, there have been substantial research studies reported on millet processing to maximize the nutritional benefit of millet. The mechanism underlying some of the processing methods remain inadequately explored due to the limited number of in-depth molecular-level research studies. One approach is to physically separate the part of the grain with concentrated antinutrient components. Another involves breaking the chemical bonds within the antinutrient functional groups, thereby generating fragmented products that no longer behave as an antinutrient. A third method is biochemical conversion, which transforms the antinutrient into a simpler compound with no antinutrient properties. Based on these principles, various processing techniques can be applied to millets to target a reduction in their antinutrient content. For instance, decortication falls under physical separation, as it removes the outer layers where antinutrients are often most concentrated [1]. Heating the grain introduces enough energy to break or dissociate bonds of functional groups responsible for antinutrient activity. Additionally, enzymatic breakdown during bioprocessing (such as germination and fermentation) can alter the characteristic bonds, converting them into compounds without antinutrient potential. Milling includes mechanical grinding and abrasion action to break down the grains into smaller fractions and remove bran and germ from the endosperm. The removal of outer layers during milling slightly reduces the concentrations of phytates and tannins. The heat generated in friction-type milling or grinding operations may also degrade the antinutrient compounds. Soaking promotes the leaching of soluble compounds (tannins and phytates) into water to reduce their concentration. Germination reduces the level of antinutrients through the activation of enzymes that break down the compounds (tannins and phytic acids) [174]. Malting reduces the levels of antinutrients due to the initial soaking and germination processes, whereas fermentation produces several enzymes (for example, phytase) capable of degrading the polyphenols and phytates and producing beneficial organic compounds. Enzymatic treatments include specific enzymes that break down the targeted compounds into simple and non-inhibitory forms and enhance nutritional quality. Parboiling is a hydrothermal process including soaking, steaming, and drying. Steaming includes heat and moisture treatment that disrupts the structure of antinutrient compounds and breaks them down into simpler forms. In addition, the water-soluble compounds leach into hot water to reduce concentration. Roasting and puffing include high temperature that results in the thermal degradation of compounds, the rapid evaporation of moisture, and the expansion of products, altering the structure and breaking down or inactivating the compounds [19]. Extrusion involves extremely high temperature, pressure, and mechanical shear, leading to substantial changes in material structure and inactivates enzymes (phytases) and trypsin inhibitors (serpins), gelatinization process enhances the digestibility of starch [175]. Microwave treatment involves a rapid temperature rise that inactivates the enzyme inhibitors; the partial cooking under heat promotes the leaching of water-soluble bioactive compounds into water and enhances the digestibility of nutrients [176].

In non-thermal processing methods, the degradation of antinutrients depends on the type of energy applied, which must be sufficient to break down the covalent or non-covalent bonds associated with these compounds. Thus, the effectiveness of non-thermal methods for antinutrient degradation can vary widely depending on the energy form used. For example, cold plasma includes the generation of reactive species (ions and radicals) that interact with the functional group of antinutrients, effectively breaking down these compounds and reducing their bioavailability [177]. Although high pressure does not impact the covalent bonds, high pressure generally disrupts the tissues or cell walls, thus releasing these bioactive compounds into water. High pressure could also disrupt the enzymes and protein structures that are linked to stabilizing the phytates and inhibitory effects. In ultrasound treatment, the shear forces generated from cavitation initiate the mechanical breakdown of antinutrients. The cavitation also promotes the release and extraction of bioactive compounds; the localized heating and pressure changes disrupt the cellular structure, causing the breakdown of antinutrients, and ultrasound treatment promotes enzymatic degradation of antinutrients and enhances the bioavailability of nutrients [178]. Ionized radiation treatment generates free radicals and reactive species that can break down bioactive compounds to reduce their concentration. The structural alteration due to irradiation reduces the chelation capability of antinutrients with minerals and proteins, thus enhancing the bioavailability. Ozone treatment promotes oxidative reactions that may break down bioactive compounds and reduce the inhibitory activity of these compounds against nutrient absorption [117]. There is a critical need for comprehensive research to better understand the mechanisms involved and their effects on antinutrient degradation. It is essential to determine the specific forms into which antinutrients are converted during degradation. This requires detailed spectroscopic analysis with precise quantification to reveal whether the degraded products are fragmented antinutrients or nutrient components. Additionally, accurately representing antinutrient concentrations is essential, particularly in processes where total mass changes, such as roasting. Expressing antinutrient levels on a dry mass basis becomes crucial to reliably determining whether true reductions in antinutrients have occurred. Furthermore, the accuracy of analytical methods requires close examination; for example, colorimetric assays often struggle to distinguish between antinutrient and nutrient phenolics and typically estimate only solubilized compounds, overseeing insoluble forms.

Throughout this review, a thorough examination of the effects of various processing techniques on individual components was performed. It is essential to recognize that these components naturally exist as a complex, heterogeneous mixture within millet seeds. Therefore, identifying a processing technique that effectively reduces antinutritional factors while simultaneously enhancing antioxidant levels is critical for potential therapeutic applications. Bioprocessing methods such as soaking, germination, fermentation, and enzymatic treatment exhibit promising results in reducing antinutrients in millets. These processes effectively degrade compounds like phytates, tannins, lectins, and goitrogens, thereby improving nutrient bioavailability and digestibility. Additionally, biological treatments enhance the antioxidant potential of millets by promoting the release of bioactive compounds such as phenolic acids, flavonoids, and peptides, which exhibit potent health-promoting properties. Thermal techniques like boiling, steaming, roasting, and extrusion have been extensively studied for their influence on millet nutrients and antinutrients. These methods not only alter the structure and digestibility of millet grains but also influence phenolic compounds and antioxidant activity. Thermal processing degrades the heat-sensitive antioxidants; however, moderate roasting has been found to enhance TPC and AOX capacity, thus balancing the trade-off between processing and nutritional quality. Non-thermal technologies, including ultrasound, HPP, and irradiation, are emerging as viable alternatives to traditional thermal methods. These methods preserve heat-sensitive antioxidants while effectively reducing antinutritional factors. For instance, ultrasound enhances enzymatic activity during germination and reduces saponins and lectin contents. Similarly, HPP and irradiation induce structural changes in antinutrients, enhancing the safety and nutritional value of millets. These findings underscore the importance of selecting appropriate processing methods tailored to specific millet varieties and intended applications. While biological methods are effective in reducing antinutrients and enhancing antioxidant activity, thermal and non-thermal techniques offer complementary benefits in preserving nutrients and reducing health effects of antinutrients. These insights are crucial for developing sustainable processing strategies that optimize millet’s nutritional benefits while meeting consumer preferences for minimally processed and health-promoting foods. The combination of multiple techniques was found to offer superior results and viable options compared to the single method. Overall, the optimal processing method to produce nutritionally valuable millet combines germination and fermentation. It is recommended to soak the millet in water for 8–12 h to activate enzymes that reduce antinutrients, followed by germination for 24–48 h, which enhances nutrient bioavailability and enhances bioactive compounds. Fermentation using natural or added starters for 12–24 h further breaks down the antinutrients and enhances the antioxidant levels. This sequence effectively reduces major antinutrients such as phytates and tannins while retaining essential nutrients, making it ideal for maximizing millet’s nutritional quality.

## 7. Conclusions

The processing methods employed in millets have profound implications for their antioxidant capacity and antinutritional factors. These methods play a crucial role in enhancing the nutritional profile of millets while reducing health risks associated with antinutrients. The integration of physiochemical, bio, thermal, and non-thermal processing methods seems to be a promising approach for enhancing the nutritional quality and safety of millets. In addition to this, the optimization of processes or process combinations to simultaneously enhance antioxidants and reduce antinutrients in the same treatment system remains a key research gap that warrants further exploration. Future research could focus on optimizing these techniques to maximize the health benefits of millets, explore non-thermal-based combination techniques, broaden the application across different millet varieties, and examine their potential synergistic effects on overall nutrition and human health. The processing techniques hold significant potential for future research and practical applications in millet processing on a large scale, with the aim to enhance the value of millet-based dietary food products.

## Figures and Tables

**Table 1:** Role of antinutrients in various health risks.

Sl. No.	Antinutrient	Impact on Health and Nutrient Availability	Type of Compounds and Mode of Action	Reference
1.	Polyphenols	Reduce the nutritional value and availability of minerals and proteins	Procyanidin compounds chelate the minerals and proteins to form complexes.	[37]
		Reduce protein digestion and availability	Interfere with protein to forms complexes that decreases the solubility and reduce proteolytic enzyme activity	[38]
		Loss of body weight, appetite, breathing, and cardiac issues	Interfere with protein absorption and forms complexes	[39]
2.	Tannins	Interfere with mineral absorption, reduce bioavailability and bioaccessibility of minerals (Ca, Zn, Fe, Mg, and K)	Chelate the metal ions, inhibit the enzyme activities, and prevent absorption of nutrients	[40,41,42,43]
		Reduces protein and carbohydrate digestion, absorption, and palatability	Forms tannin-protein complexes, inactivates digestive enzymes, thus reduces protein digestion and absorption	[44,45]
		Digestive disorders	Type—condensed tannins binds and shrinks protein. Inhibits the action of digestive enzymes like trypsin, chymotrypsin lipase, and α-amylase	[46]
3.	Phytates/phytic acid	Interfere with minerals (Fe, Zn, Ca) absorption and bioavailability	Phytates chelates with di- and trivalent minerals Forms mineral complexes that cannot be digested or absorbed	[40,42,43,46]
		Interfere with mineral absorption, solubility, functionality, palatability, protein, and carbohydrate digestion	Due to inhibition of digestive enzymes like pepsin, α-amylase, and trypsin. Phytate indirectly reacts with proteins and makes them undigestible. In absence of phytase, phytic acid binds the proteins.	[42,46]
4.	Phenolic acid	Reduce the mineral availability	Chelating metal ions like Fe and Zn	[37]
5.	Saponins	Reduce nutrient (Vitamins A and E and lipids) absorption, interfere with integrity of epithelial cells, thyroid and gut functions	Due to inhibition of metabolic and digestive enzymes Binds the minerals	[40,45]
6.	Oxalates	Kidney stones and protein indigestion	Interfere with Ca and Mg metabolism and their absorption Forms complexes with protein and inhibits peptic digestion	[40,45]
7.	Trypsin inhibitors	Protein indigestion	Due to loss of trypsin and chymo-trypsin in gut, the digestion process is affected	[29,45]
8.	Protease and amylase enzyme inhibitors	Inhibit growth, can cause pancreatic hypertrophy, reduce protein digestibility	Protease inhibitors will bind proteins and reduce the growth Protease inhibitors can also increase digestive enzyme secretion in pancreas	[40,47]
9.	Dietary fiber	Barrier for digestion, delayed nutrient absorption, increase fecal transit time	High fiber content in food can lead to several intestine disorders	[1]
10.	Lectins and haemagglutinins	Cause impaired growth, reduced nutrient absorption, inflammation, leaky gut syndrome, affects pancreas function	Can bypass human defense mechanism and break intestine surface. Can stimulate WBC to cause autoimmune disease	[29,42,45]
11.	Oxalates	Inhibit Ca absorption, increase risk of kidney stones (Ca)	Chelating the Ca, reducing its absorption in the intestine and colon	[42]
12.	Goitrogens	Causes hypothyroidism and goiter	Glucosinolates and thiocyanate may have adverse effects on the thyroid Due to interference with iodine uptake	[42]

**Table 2:** Concentration range of phytochemicals, antinutrients, and antioxidant activity in whole millet or flour fraction of different millet varieties.

Compounds	Pearl Millet	Foxtail Millet	Proso Millet	Little Millet	Kodo Millet
TPC (mg GAE/g; d.w)	1.29–4.79	0.22–1.29	1.14	1.51	4.44
Total free phenolic acids (mg/g; d.w)	0.098	0.084	0.071	0.053	0.172
Total bound phenolic acids (mg/g; d.w)	1.04	0.306	0.312	0.166	1.4
TFC (mg QE/g or CE/g; d.w)	4.9–6.2 * QE	6.28–7.61 QE	3.34 QE	1.18–3.34 CE	1.01–1.87 CE
α-amylase inhibition of free phenolics (µg GAE/mL)	122.59	135.3	99.48	98.77	125.8
α-glucosidase activity of free phenolics (µg GAE/mL)	82.4	87.92	78.91	81.15	92.61
DPPH RSA activity (%)	24.8–73.7	25.3–73.5	26.87	23.0–34.1	53.1–56.7
FRAP RSA activity (mM Fe (II)/g)	0.22	0.19	0.18	0.16	0.21
ABTS RSA activity (%)	62.8–90.6	36.95	55.43	31.21	60.75
Total tannins (mg/g; d.w)	0.239	0.287	0.218	0.157	0.48
Phytic acid (mg/g; d.w)	4.7–9.2	5.91–9.73	5.2–9.3	6.3–8.3	1.2–8.3
Saponins (mg/g; d.w)	38.6	8.73 *	-	-	-
Oxalates (mg/g; d.w)	0.26–0.36	0.119	0.085	-	0.034

Note: * Refers to whole millet flour fraction, TPC is total phenolic content, TFC is total flavonoid content, GAE is gallic acid equivalents, CE is catechin equivalents, QE is quinone equivalent, FAE is ferulic acid equivalent, RUE is rutin equivalent. Source: [29,35,36,37,46,48,49,50,51,52,53,54,55].

**Table 3:** Summary of physiochemical and bio processing techniques and their influence on antioxidants and antinutrients in millets.

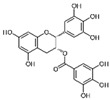	Physiochemical and Bioprocessing Techniques						
	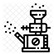	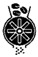	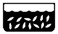	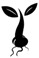	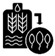	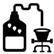	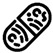
Phytochemical Compounds	Decortication	Milling/Dehulling	Soaking	Sprouting/Germination	Malting	Fermentation	Enzymatic Treatment
Total phenols	D	D	I or D	I or D	I or D	I or D	I or D
Total flavonoids	D	D	D or N	I or D	I or D	I	I
Tannins	D	D	D or N	I or D	D	I or D	I or D
Phytate	D	D	D	D	D	D	
Saponins			D	I or D		D	
Oxalates		D	D	D		D	
Dietary fiber	D	D		I or D	D	I or D	D
Trypsin & amylase inhibitor			D	D		D	
Lectins	D	D	D	D		D	
Lignans	D	D					
Carotenoids	D	D	D				
Total anthocyanins							
Antioxidant capacity	D	D	I or D or N	I	I or D	I	

Note: D—decreases, I—increases, N—not altered, empty cell—data not available.

**Table 4:** Summary of thermal and novel non-thermal processing techniques and their influence on antioxidants and antinutrients in millets.

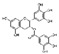	Novel Non-Thermal Processing Techniques						Thermal Processing Techniques							
	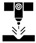	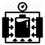	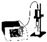	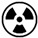	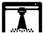	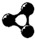	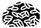	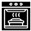	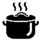	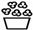	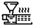	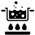	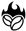	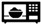
Phytochemical Compounds	Cold Plasma	High Pressure Processing	Ultrasound	Gamma Irradiation	Electron beam Irradiation	Ozone Treatment	Parboiling	Baking	Steaming	Popping/Puffing	Extrusion	Boiling/Cooking	Roasting	Microwave Treatment
Total phenols	I or D	I	D	D	D	I	I	I	I or D		I or D	D	I or D	D
Total flavonoids	I or D	I	I or D	I		D			I or D or N			D	I or D	I or D
Tannins	D	D	I or D	D	D	D			I or D	D	D	D	D	I or D
Phytate	D	D	I or D	D	D	I	D	D	D or N	D	D	D	D	I or D
Saponins	D		I or D											I or D
Oxalates				D				I		D		D	I or D	
Dietary fibre	D						D	D	I or D	I	D	D	D	
Trypsin & amylase inhibitor								D		D		D		
Lectins								I	D			D	I	
Lignans														
Carotenoids	D					D					D		D	
Total anthocyanins												D		
Antioxidant capacity	D		I or D	I		D	I	I or D	I		I	D	I or D	I or D

Note: D—decreases, I—increases, N—not altered, empty cell—data not available.

**Table 5:** Summary of the effect of physiochemical and biological methods and their influence on antinutritional compounds.

Processing Method	Processing Conditions	Results and Important Observations *	Inference	References
Solid state fermentation (SSF)	Pearl millets soaked at 30°C for 12 h, autoclaved at 121°C for 15 min, and fermented with *Rhizopus azygosporus* culture for 10 days at 25 °C.	TPC of fermented pearl millet (FPM: 6.58 mg GAE/g) was higher than unfermented pearl millet (UFPR: 21.78). Condensed tannins enhanced from 101.3 to 176.7 md CE/100 g for 10 days FPM. α-amylase (IU/gds), β-glucosidase (4–86), and xylanase (7–77) activities were increased from 61–201, 4–86, and 7–77, respectively, for 10 days FPM. Similarly, DPPH, ABTS, and total antioxidant capacity were also increased from 89.3 to 95.5%, 97.8 to 99.1, and 20.1 to 29.2 mg AAE/g, respectively.	Fermentation significantly enhanced bioactive compounds such as TPC, condensed tannins, and enzymatic activities. Production of several enzymes during SSF, which break down the complex bioactive to enhance the TPC. Antioxidant activity of FPM was 16.4 times higher than UFPM due to increase in enzymes during SSF.	[56]
Decortication	Pearl millets were decorticated using stone dehuller (800–1200 rpm) or modern abrasive dehuller.	Tannins (mg/100 g) in whole millet (343.9) were reduced to 174.4 and 230.5 for modern and traditional decortication method, respectively, for white cultivar. Similar trend was recorded for green millet cultivar. Phytic acid (mg/100 g) in whole white cultivar (301.1) was also reduced to 77.5 (modern) and 98.9 (traditional); a similar range of reduction was seen for green cultivar.	In addition to decortication, the storage period (0 to 60 days) also reduces the tannins (343.9 to 162.9) and phytic acids (301.1 to 256.8) in processed and unprocessed grains. Modern decortication significantly reduced the tannins (in both cultivars) and phytic acids (only in green cultivar) compared to traditional method. Complete removal of hull or bran and peripheral parts during decortication leads to reduction in tannins and phytates.	[57]
Soaking and germination	Finger millet was soaked for 12 h at 30 °C, germinated for 12, 24, and 36 h at 30, 34, and 37 °C and 80% RH, dried at 60 °C for 6 h, and milled (100 mesh size).	Oxalic acid (mg/100 g) was reduced from 118 to 96 at 12 h soaking, further to 53.8 at 16 h germination at 25 °C. Phytic acid (mg/100 g) in raw (676.7) was reduced to 587 at 12 h soaking, further to 238.4 at 36 h germination at 37 °C. Trypsin inhibitor activity of raw (6.59 TUI/mg) was reduced to 5.7 at 12 h soaking, further to 1.9 at 36 h germination at 37 °C.	During soaking and germination, the oxalic acid and phytates leach into water; also, the phytase enzyme is activated during germination, which reduces the concentration. Changes in endosperm and axis of millets during soaking and germination alter the trypsin activity. Highest decrease in phytic acid and trypsin inhibitor was recorded at 36 h germination at 37 °C.	[58]
High pressure soaking and germination.	Foxtail millet germinated and non-germinated samples were subjected to high pressure of 200, 400 and 600 Mps at 20, 40, 60, and 80 °C for 30, 60, 90, and 120 min duration. Treated samples were dried to 10% moisture and milled (150 µm sieve).	Optimum condition for high-pressure processing were: 400 MPa, 60 °C, and 60 min soaking time. Germination increased the TPC by 77.4%. The phytic acid and tannin contents were reduced by 39 and 45%, respectively, due to the action of phytase enzyme during germination and leaching during soaking. FRAP AOA was also increased by pressure, temperature, and soaking treatments.	Due to increase in water uptake at all soaking pressures and temperatures, hydrolytic enzyme were activated which broke down the cell wall. Increase solubility of phytic acid complexes during high pressure and temperature treatment degraded the phytates. Thermal degradation and leaching of water soluble reduced the tannins. High-pressure treatment disrupts the cell walls and organelles and improves the antioxidant extraction.	[59]
Soaking, malting, and popping	White finger millet varieties. Soaking (12, 24, and 48 h) and dried at 65 °C. Malting: steeped overnight, germinated at 25 °C (12, 24, and 48 h) and dried at 60 °C for 6 h Popping: Conditioned (19% M.C) and popped at 170–200 °C.	Soaking for 0–48 h reduced the initial phytate (266 mg/100 g) by 16.9%. Malting for 12 and 28 h linearly reduced the phytate by 11.7 and 50.6%, respectively. Popping also reduced phytate by 17.1% from the initial value of 266 mg/100 g.	The highest average reduction of phytate was recorded for 48 h malting (266–132 mg/100 g) followed by popping (266–221 mg/100 g) and 48 h soaking (266–221 mg/100 g) for different varieties. Soaking, malting, and popping with increased intervals enhances nutrients by inactivating anti- nutrients and also improves the carbohydrate and protein digestibility.	[60]
Malting	Brown (BFM) and dark brown finger millets (DBFM) soaked for 24 h at 25 °C, sprouted, kilned for 8 h at 50 °C, milled. Malting duration 0–96 h.	Decrease in TPC was recorded for DBFM malt (4.4–4.3 mgGAE/g) at 96 h, while in BFM malt (7.0–4.4 mgGAE/g) it was at 24–48 h; later, the TPC increased (4.4–5.2 mgGAE/g) at 72–96 h malting. Protocatechuic acid, epicatechin, and catechin were predominant phenolics reduced. TFC of DBFM and BFM were found to reduce 0.92–0.35 mgQE/g and 5.4–2.03 mgQE/g, respectively, with increase in malting time 0–96 h. Malting did not alter DPPH RSA of BFM, while it increased (51–70.1%) at 24 h malting of DBFM. ABTS antioxidant capacity (µM TE/g) slightly reduced (10.3–9.8) for DBFM at 96 h and increased (9.7–10.6) at 72 h, respectively.	Study revealed the presence of higher flavonoids than phenolic acids in finger millet malt than unmalted sample. TPC are concentrated at 24 h malting, and millet varieties influence the phenolic compounds (PC). Oxidative degradation, bioconversion of PC during malting, and leaching of hydrolysed of during steeping are known to reduce the PC. Increased endogenous enzymatic hydrolysis on bound phenolics increased PCs.	[61]
Germination and malting.	Sorghum grains steeped for 6 h and sprouted at 32 °C for 0, 48, and 96 h.	Phytate (459–1097 mg/100 g) and tannins (0.12–4.4 mg/g) were reduced by 32.7–56.3% and 13.2–50%, respectively, after 48 h germination.	After 96 h germination, tannin reduction was further increased by 26.3% to no detection level. Malting can eliminate the phytate and tannin in sorghum at 96 h of germination.	[62]
Enzymatic treatment	Finger millet decorticated, milled (60 µ sieve). Xylanase and Cellulase enzymes at different concentrations added and incubated at 50 °C for 2 h, deactivated at 90 °C for 5 min, decanted, and lyophilized. Samples were extracted at various temperatures, time, and solvent concentrations using ultrasound-assisted extraction.	The TPC increased with increase in enzyme (xylanase, cellulase) concentration (up to 1500 U) with and without sonication treatment, after which phenolics reduced. Among the two enzymes, xylanase offered 20% higher yield (47.9 g/100 g) than cellulase sample (37.7 g/100 g). The flavonoids (mg/100 g) obtained from sonicated (492) and xylanase treated + sonicated sample (483) were higher than heat reflux sample (350). Tannin content (mg/100 g) was highest (915), followed by sonicated (850) and heat reflux (725) sample.	High TOC yield xylanase sonicated sample was due to high content hemicellulose covalently linked with ferulic acid bridges. The difference in tannins and flavonoids was due to solubility, solvent polarity, and thermal degradation of phenolics during heat reflux. Heat treatments can significantly reduce the tannins and flavonoids.	[63]
Decortication & milling	Sorghum millet was decorticated in a rotary mill, polished (9, 15 min), and sieved BSS 36 mesh.	Dietary fiber of whole grain (35.2%) decreased to 20% in 15 min polished grains. Whole grain condensed tannins (5.5 mg/g) reduced to 0.5 mg/g in polished grains. Whole grains total phytate (18 mg/g) reduced to 16 mg/g in polished grains.	Decortication and polishing of grains for 15 min resulted in >50% loss of fiere, >80% reduction in tannins, and >30% reduction in phytates.	[64]
Fermentation	Pearl, finger, foxtail millets. Three methods of fermentation. 1. FPM1: unground finger millet flour fermented for 72 h at 30 °C with 0.2% *S. boulardii* and spray dried. 2. FPM2 and FPM3: Mixing ground finger millet four and inoculated with combination of yeast and LAB (*S. cerevisiae* + *C. paraminentarius*, and *G. uvarum* + *F. sanfranciscensis*) and fermented for 24 h at 28 °C.	Pearl millet had the highest (0.3–0.74) free phenols (mg/g), followed by finger (0.3) and foxtail (0.26) millets. The free phenolics in FPM1, FPM2, and FPM3 fermented samples were 0.12, 0.27, and 0.25, respectively, and were lesser than unfermented finger millet (0.3–0.76). Total phenols (2.26 mg/g) in unfermented pearl millet by acid hydrolysis were reduced in FPM1 (1.2) and increased in FPM2 (2.74) and FPM3 (2.57).	Fermentation with single culture or mixture of culture both reduced the free phenolics and total phenols. In all treatments, the mineral concentration was enhanced. Fermentation with lactobacilli and yeast among all other strains significantly enhanced the nutritional value and nutraceuticals compared to the unfermented samples.	[65]
Malting and fermentation	Sorghum grains malted for 3, 4, and 5 days, drying at 65 °C for 6 h, hammer mill (2 mm), malt extraction (72 °C for 12 h), and 7 days fermentation at temperatures 20 and 35 °C to develop beverages.	Raw sorghum and finger millet composite flour containing 105 mg/100 g tannin and 0.83 GAE/100 g total phenolics was reduced to a range of 1.29–40.08 mg/100 g and 0.13–1.66 GAE/100 g, respectively, in different beverage formulations. The increase in malting days from 3 to 5 days increased the phenolics (0.35 to 0.92) and decreased the tannins (40 to 1.74).	Tannins initially increased due to hydrolysis of condensed tannins and later reduced due to action of phenyl oxidase or binding with cotyledon, making them undetectable. Phenolic enhancement was attributed with increase in free phenolics due to hydrolysis of bound phenolics by hydrolytic enzymes.	[66]
Fermentation with three LAB species	Sorghum flour fermented by three LAb (*L. brevis*, *L. bulgaricuss*, *L. casie)* with and without NaOH immersion and combination treatment.	Combined fermentation with NaOH immersion treatment offered the best reduction of phytic acid (11.9 to 0.11%), tannins (6.16 to 0.063%), and enhanced the protein availability (8.5 to 14.6%).	There was no significant difference observed between fermentation with different LAB species on phytic acid and tannin reduction. Combined fermentation technique may be feasible as alternate technique for substitution of wheat in flour production. The optimized method also reduced the glucose level (2.7–1.83%) and glycemic index (40.7–36.3) of modified flour.	[67]
Malting	Pearl millet steeped for 24 h at 30 °C, germinated at 34 °C for 24 h, sprouted for 48 h, dried in cabinet dryer at 60 °C for 5–6 h, and disc milled.	Malted millet had flavonoids (48.4 mg/100 g) higher than unmalted wheat flour (19). The AOC of malted millet was the highest, 49.3 mg/100, compared to wheat flour (15.1). The DPPH RSA of malted millet was 83.5%, slightly less than wheat flour (87%).	The higher phenolic content in malted millet was due to hydrolysis of condensed procyanidins. Flavonoids serve as potent antioxidants and free radical scavengers that create oxidative stress on cells. Malted millets serve as good dietary source of antioxidants and ingredients for functional foods.	[68]
Fermentation	Pearl millet Rabadi prepared by soaking and fermenting in butter milk at 30, 37.5, 45 °C for 5, 10, and 15 h, course grinding (15 s), cooked with fermented sample, molded, and dried at 50 °C for 12 h.	Phytic acid (762–772 mg/100 g) reduced by 17.8–18.9% at all soaking periods as temperature increased from 30 to 45 °C.	Fermentation due to low pH of dough reduced the phytate due to phytase enzyme in both cultivars. Natural fermentation at higher temperature (45 °C) significantly reduces the phytate compared to low temperature (37.5 and 30 °C).	[69]
Germination and Fermentation	Pearl millet steeped (14–24 h), germinated (3–4 days), sun dried (2–3 days), homogenized (200 mm sieve), and malted.	Oxalates (mg/100 g) of raw millet (4.86) decreased in sprouted (3.87) and fermented (2.88) samples. Tannins in raw (0.5 g/100 g) were equally enhanced in both germinated and sprouted samples (0.68). Phytate in raw (15.7 mg/100 g) > sprouted (10.7) < fermented (18.1) sample.	Fermentation can polymerize catechins into tannins. Fermentation and germination activates α-amylase and maltase enzymes to degrade starch into sugars and maltodextrins. Phytate and phytate phosphorus were low, in range of 0.5–1.03, indicating high bioavailability of phosphorus.	[70]
Fermentation	Pearl millet fermented at 38 °C for 0, 24, 48, 72, and 96 h using mixture of *L. pentosus*, *L. sanfranciscensis* and yeast strains.	Total phenols in basic hydrolyzed unfermented (149.3 mg/100 g) were lesser than fermented sample (206 mg/100 g), which were increased after acid hydrolysis to 193.6 and 278.3 mg/100 g, respectively. The initial free phenols (126.9 mg/100 g) were increased to 145.3 mg/100 g at 72 h fermentation and were unaltered after 96 h.	Free phenols ranged from 64–127 mg/100 g, and 68–87% were flavonoids. A total of 12 phenolic compounds were identified. Action of microbes using fermented and soluble fiber released the phenolic compounds in the fiber, which enhanced (36%) the total phenols during fermentation.	[71]
Soaking and sprouting	Millets: little, foxtail, pearl, finger, and kodo millets. Soaking for 24 h and sprouting for 24 h.	The tannins (mg/g) in raw millets were in the following order: 1.2 (little) < 1.4 (kodo) < 1.8 (pearl) < 1.9 (foxtail) < 2 (finger); was reduced after soaking and sprouting in the following order: little (0.34) < kodo (0.52) < pearl (0.53) < finger (0.55) < foxtail (1.2) millets. Phytase activity (unit/kg) was highest in the following order: kodo, little, finger, foxtail, and pearl millet. The levels were increased in the following order: little > kodo > foxtail > finger > pearl millet. Saponin was initially lowest in kodo < finger < little < foxtail < pearl millet and increased to highest in pearl > finger > little> foxtail > kodo millet. After soaking and sprouting, the DPPH activity was highest in the following order: 81% (foxtail), 78.3% (kodo), 62.4% (pearl), 56.1% (little), and 55.5% (finger) millet.	Tannin reduction was highest in finger millet (73.3%). The highest saponin content was in soaked and sprouted pearl millet (39.53 mg/g). After prolonged soaking and sprouting, the phytase activity was still highest in little millet (61.52 µg/kg). Sprouted foxtail millet was found to have antioxidant activity (81.13%). During soaking and sprouting, the endogenous and newly synthesized enzymes alter the grains to enhance the saponins. Release of bound phosphorus during soaking and germination enhances and triggers the phytase activity.	[35]
Spontaneous fermentation	Light and brown variety of finger millet milled (2 mm sieve), spontaneously fermented at 37 °C from 0 to 96 h, and oven dried (45 °C for 24–48 h).	Prepared fermented porridge and assessed the bioactive and AOA. TPC in untreated light and brown flour were increased from 166 to 488 mg/100 g and 420 to 606 mg/100 g, respectively, in 96 h fermented porridge. Fermented light and dark drown had increased TFC from 160–330 mg/100 g and 249–466 mg/100. TAC significantly reduced from 58–32 mg/100 g (light brown) and 69–38 mg/100 g (dark brown) with increase in fermentation time from 0 to 96 h due to action of glycosidase to hydrolyze anthocyanins in cotyledon. DPPH RSA increased from 52 to 58% (light brown) and 53 to 59% (dark brown) for 0-96h fermentation The increase was due to the release and synthesis of bioactives from cell walls.	Variations in TPC may be due to action of enzymes producing free phenolics from bound phenols, accumulated phenols in seed coat, and varietal difference. Enzymes like amylase, glucosidase, phytase, tannase, lipase, cellulase, and inulinase formed during fermentation play a vital role in breakdown of the cell wall and release flavonoids and free phenolics, which enhance the AOA. Dark brown has more anthocyanins than light brown varieties. Fermentation enhances the AOA by releasing flavonoids and create simple phenolics by the action of LAB.	[72]
Natural fermentation	Finger millet fermented (72 h at 28 °C) using LAB species (*B. cereus*, *L. plantarum*, *L. casei*, and *L. brevis*), and dried (50 °C for 48 h).	Increase in fermentation time (0 to 72 h), the reduction of initial antinutrients like oxalates (1.5–0.87 mg/100 g), trypsin (6.2–2.7 mg/100 g), phytate (0.85–0.19 mg/100 g), and tannins (3.2–1.5 mg/100 g) was noted for 72 h fermented samples. Total flavonoids enhanced (63.3–172.8 mg/100 g), while total phenols (40.2–11.6 mg/100 g) and antioxidant potential (68.2–28.1 mg/100 g) were reduced with an increase in fermentation time from 0 to 72 h.	TPC decline was associated with factors like grain types, microbe species, and conditions like pH, temperature, and time. Reduction of phenolic compounds with fermentation time also reduced the AOX potential.	[73]
Germination	Foxtail and little millets germinated at 35 °C and 30 °C for 24 h, respectively, overnight steeping, tray dried (60 °C for 6 h), and milled.	Millets soaked (12 h), germinated (24 h), dried and milled. TPC (mg GAE/100 g) of whole foxtail was enhanced (55.9 to 69.2), while reduced slightly in finger millet (132 to 123.3) after germination. TFC (mg RE/g) increased in both whole foxtail (12 to 12.4) and little (7.4 to 10) millet after germination. Tannins of whole foxtail (4.9%) and little (6.1%) millet were increased to 6.2 and 9.1%, respectively, for germinated flour. Similarly, oxalates (g/100 g)) of whole foxtail (0.56) and little (0.44) were reduced to 0.37 and 0.37%, respectively, post-germination. DPPH RSA of germinated flours were higher than whole flour in both millets. Germinated foxtail RSA was slightly higher (3.6 mg/mL) than germinated little millet (1.9 mg/mL).	Enhanced RSA in both millet flours due to breakdown of high-molecular-weight polymers. Oxalate increase was due to activation of oxidase and its action on oxalic acid to form CO_2_ and H_2_O_2_. Tannins are mostly present in seed coats and are less affected by germination. TPC are increased due to release of bound phenols through breakdown of cell wall by the action of enzymes.	[5]
Germination	Finger millet soaked overnight, germinated at 25 °C for variable time of 12 to 96 h, dried at 50 °C, and milled to flour.	TPC of non-germinated (1.5% GE) reduced to 0.49% GE after 24 h germination, later increased to 1.13% during prolonged time (96 h). Similarly, the tannins (173.7 mg/100 g) initially reduced to (64.3) after 48 h and increased (94.6) after 96 h of germination. The phenol-dependent antioxidant activity initially decreased from 72.2–57.7% and increased to 84.4% with germination time.	Decreases of TPC and TTC were due to leaching of polyphenols and increased enzymatic action. The increase in seed coat (%) in later germination stages due to loss of carbohydrates increased the phenols during prolonged germination. The variation of AOA was due to variation in TPC and ascorbic acid with germination time. A prolonged germination (>72 h) must be avoided as it results in high malting loss and increase in antinutrients.	[41]
Ultrasound assisted hydration	Finger millet hydrated by ultrasound treatment with different variables like amplitude (30–70%), time (10–20 min), and grain-water ratio (1:3 to 1:6).	Assess the effect of ultrasound on hydration rate and reduce antinutrients. An ultrasound treatment (66%) for 26 min with grain-water ratio of 1:3 resulted in the highest reduction in phytate (66.9%) and tannins (62.8%). The lowest (70%, 20 min) and highest g (30%, 10 min) TPC were 190.9 and 308.8 mg GAE/100, respectively. The increase in water content significantly reduced the tannins (187 to 35 mg TAE/100 g). The phytates reduced (486.2 to 185 mg/100 g) with an increase in amplitude and soaking time.	Conversion of hydrolysable tannic-acid into gallic acid and leaching of condensed tannins reduce tannins. Heat generation at high amplitude leads to chemical degradation of phytate. Low TPC and tannins were observed in conventionally hydrated millet compared to ultrasound hydration. Conversion of tannic-acid to gallic acid and lower leaching of condensed tannins during sonication resulted in high tannins and TPC in US hydration samples.	[74]
Fermentation	Peral millet fermented using yeast. Temperature (30–45 °C), yeast concentration (2–4%), time (18–24 h).	The optimized condition was 2% yeast, 30 °C, and 18 h fermentation. Among various treatments, TPC ranged from 32.74–46.43 μg GAE/mg and TFC from 2.24–9.46 μg CAT/mg.	Low yeast (2%) and high temperature (45 °C) yielded the highest TPC. Variation of phenolics and flavonoids was due to pH variation between treatments and enzymatic action on the cell wall to release phytochemicals. Catechin, ferulic, cinnamic, and vanillic acids were predominant phenolic compounds identified.	[75]
Soaking, germination, fermentation, and microwave heating.	Pearl and finger millets. Soaking (ST) at 28 °C Germination (GT) at 25 °C for 48–72 h Microwave treatment (MT) (900 W, 2450 MHz) for 40–100 s, followed by oven drying (at 55 °C overnight) and milling using cabinet flour mill (16 mesh sieve). Open fermentation (OF): for 48 h using dried and pulverized flour and oven drying at 55 °C for 24 h. Closed fermentation (CF): Milled raw flour autoclaved at 121 °C for 15 min, inoculation (*Aspegillus niger*), fermented at 29 °C for 48 h, and finally oven drying at 55 °C for 24 h to fungal growth.	The tannins and phytic acid in both untreated (UT) millets were significantly reduced by all treatments. Tannin was least reduced (38–45%) in MT or soaking treatment, while maximum reduction (95–96.5%) was in OF or CF of both millets. For phytic acids, CF consistently offered the highest reduction range (76–86%), while soaking or MT offered the least reduced (26–27%) in both the millets. Germination of both millets had the highest reduction of polyphenols (60–78%). AOA was highest (59–66 TE g/g) for soaking treatment, which least reduced (22–24%) the polyphenols; however, it was lower than the untreated millets (77–86.8 TE g/g).	All processing treatments had a significant contribution in reduction of antinutrients and enhancement of crude protein and fiber of millet flour but affected crude fat quality. Almost 90% of tannins and 80% of phytic acids were minimized by fermentation compared to other treatments. Apart from reducing the antinutrients, treatments also negatively influenced the polyphenol antioxidant capacity with the following pattern: UT > CF > OF > ST > MT > GT. Fermentation seems to be the best processing method among all the methods evaluated for household processing to minimize antinutrients.	[76]
Sprouting and fermentation	Pearl millet sprouted (28 °C for 2, 3, 4, and 5 days) and fermented for 0, 24, 48, 72, and 96 h, followed by oven drying at 80 °C for 24 h and milled (disc mill) into flour (0.05 mm).	Sprouting and fermentation significantly enhanced the TPC, TFC, and AOA of pearl millet. Pearl millet: Increased sprouting time (0 to 96 h), linearly decreased TPC (21.1–16.25 mg/100 g), and linearly increased TFC (6.3–7.8 mg/100 g), FRAP (29.6–32.1 mM/100 g), and DPPH RSA (34.6–41.0 mM/100 g). Similarly, increased fermentation time (0–96 h), linearly increased TPC (20.1–25.6 mg/100 g), TFC (6.3–8 mg/100 g), FRAP (29.6–31.4 mM/100 g), and DPPH FSA (34.6–38.8 mM/100 g).	Prolonged sprouting and fermentation time had significant influence on enhancement of TPC and TFC, reducing power, and AOX capacities of both the millets studied.	[77]
LAB & yeast fermentation	Finger millet flour with water was autoclaved at 121 °C for 15 min, inoculated and fermented for 12, 24, and 36 h, and oven dried at 50 °C.	Unprocessed finger millet had 1.07 mg GAE/g TPC, 74.3% DPPH% inhibition, 1.64 mg/g tannins, and 6.29 mg/g phytic acids. Fermentation with LAB, yeast, and combination treatment with and without (NH_4_)_2_SO_4_ all treatments enhanced the TPC with LAB treatment being highest. AOA was also enhanced by all treatments with yeast treatment being highest. Phytic acids were reduced (by 20.8–67%) in all treatments and highest by L. brevis treatment. Tannins were also reduced (by 53.6–56%) with fermentation time in all treatments, while there was a significant reduction difference between yeast, LAB, and combined treatment.	Fermentation could serve as an inexpensive and potential method to improve the nutritional and bioactive compounds, and AOZ capacity by removing the antinutrients. Increased phytase activity and hydrolysis of polyphenolic compounds by polyphenol oxidase are associated with the reduction in phytic acid and tannins, respectively.	[6]
LAB & yeast fermentation	Foxtail millet flour with water autoclaved at 121 °C for 30 min, inoculated and fermented for 12, 24, and 36 h, and overnight dried at 50 °C.	Raw foxtail had phytic acid (591 mg/100 g), TPC (1.29 mg GAE/g), tannins (0.48 mg/g), and %DPPH inhibition of 73.5%. Fermentation with *L. brevis* for 12 to 36 h reduced phytic acid (325–314 mg/100 g) and tannins (0.28–0.23 mg/g), while it increased TPC (1.66–1.88 mg GAE/g) and AOA (78.2–79.1%). Similar trends were noted for 12, 24, and 36 h fermentation with yeast (*S. cerevisae*, *S. cerevisae* + (NH_4_)_2_SO_4_), LAB (*L. plantarum*, *L. brevis* + *S. cerevisae*).	The influence of fermentation, duration, yeast, LAB, and combined treatments on bioactive compounds alteration in finger millet flour was evident in each condition. Release of phytochemicals by breakdown of cell walls resulted in an increase of TPC and AOA.	[48]
Malting	Finger millet was steeped for 12–16 h, germinated for 24–36 h, shade dried (35 and 48 °C for 48 h), milled, and malted.	Malting reduced phenols by 50%, flavonoids by 41–84%, and tannins by 50–80% after 24 and 48 h of malt. The DPPH FRSA increased rapidly from 63–94% for 24 h malt. The AOA dropped to a range of 45–65% after 48 h malt. White finger millet had lower (355 mg/100 g) phenol contents than brown finger millet (204 mg/100 g).	Release of bound polyphenols and flavonoids through breakdown of cell wall altered the phenol levels. Malting activates endogenous enzymes to convert phenolics and leaching of hydrolyzed phenolics during steeping and germination. Hydrophobic interaction of enzymes and proteins reduces tannins. Release of minerals and organic compounds by TPC enhanced the AOA.	[78]
Soaking	Finger millet soaked at temperatures of 30, 40, and 50 °C between 0 and 24 h durations. Soaked sample dried at 50 °C and milled with 100 µm sieve.	Oxalates, trypsin, phytate, and tannins were high in the unsoaked sample. Soaking reduced all antinutrients by 40 to 50% at all temperatures and durations. TFC (66.3 to 103.9 mg/100 g) and DPPH FRSA (by 1 to 4%) increased while TPC decreased (40.2 to 16 mg/100 g) after soaking at three temperature and time combinations.	Reduction of oxalates is due to leaching and enzymatic activity of oxalate oxidase. Soaking can reduce trypsin inhibitors by their inactivation or loss of function.	[79]
Steeping, fermentation, germination, and combination treatment.	Pearl and finger millet steeped in water (28 °C for 16 h), dried, fermented for 72 h, germinated for 12 h, try dried at 55 °C, and hammer milled.	Phytic acid was reduced by 42% and 24% (steeping), 54% and 34% (fermentation), and 70% and 75% (germination) for pearl millet and finger millet, respectively. Tannins reduced 20% and 38% (pearl) and 13% and 37% (finger), respectively, after fermentation and germination. TPC reduced by 14% and 43% and TPC by 57% and 36% for pearl and finger millet after fermentation and germination. Steeped flour offered better DPPH FRSA, followed by germination of pearl millet, hile in finger millet, both processing methods offered better DPPH FRS than the unprocessed sample.	The alteration of bioactive was mainly due to leaching and phytase activity. Prolonged hydration reduces the total phenolic compounds and AOX activity. Germination and fermentation reduce TPC significantly and antioxidant capacity.	[80]
Soaking, germination and fermentation and combination treatment.	Proso millets soaked (12 h), germinated (48 h), fermented (20 h), and combination treatment of all three methods.	Tannins reduced (73.4 to 26.5 mg TA/100 g) phytic acid (8.77 to 2.4 mg/g) and saponins (167 to 29.1 mg/100) after all three treatments to raw millet flour. Combination of germination and fermentation results in maximum reduction in tannins and phytic acids. Saponins showed a different trend; the highest reduction was seen for soaking.	Soaking leaches phytates and tannins into water while germination activates the phytase enzyme to degrade phytate. Leaching and oxidative degradation with enhanced polyphenol oxidase reduces tannins.	[81]
Traditional fermentation	Pearl and finger millets soaked for 4 h, course grinded, and sieved (0.25 mm). Flour was added to boiling water and cooked, cooled, and dumplings were formed. Dumplings were mixed with water or cured and fermented overnight.	Phytic acids reduced by 16.1 to 49% in all millets after cooking and 20.9 to 54.5% after fermentation with or without curd. A maximum reduction of 3.75 mg/g for finger and 3.37 mg/g for pearl millets was recorded.	Fermentation with curd activates phytase enzyme, which hydrolyzes the phytic acids and converts into myo-inositol and inorganic phosphate.	[55]
Fermentation	Foxtail millet fermented (0 to 48 h) using lactic acid bacteria (LAB) extracted from fermented cabbage. Fermented grains were dried, disc milled, and sieved (100 mesh size).	Increase in fermentation time (0 to 48 h) decreased the phytic acid (5.33 to 1.78 mg/g), tannins (2.7 to 0.85 mg/g), total phenolic (7.4 to 9.06 mg GAE/g), and increased antioxidant activity (42.5 to 77.3%).	Phytase enzyme during fermentation hydrolyzed phytic acids into inositol, ortho phosphate, and/or phosphate. Proteolytic enzymes hydrolyze protein-tannin complexes to decrease the tannins. Hydrolysis of tannins also degrades the polyphenolic compounds to reduce the phenolic content. β-glucosidase enzyme from LAB hydrolyzes the phenolic glycosides to release free phenolic compounds, offering high antioxidant activity.	[82]
Steeping, fermentation, and germination	Sorghum subject in steeped for 16 h, dried. Natural fermentation: steeping for 72 h and drying. Germination: steeping for 12 h, sprouted in muslin cloth for 3 days at 30 °C. All grains dried at 55 °C for 8 h to final moisture of 8% before milling in hammer mill.	Tannins and phytic acids rapidly reduced by 34% and 23% during first 24 h fermentation and by 41% and 38% after complete fermentation of 72 h. Steeping reduced phytic acid by 21% and germination by 46%, while tannins were reduced by 52% in germinated flour. Steeped flour offered the highest DPPH FRSA, followed by fermented, while steeped-germinated flour offered the highest hydroxyl RSA. TPC reduced by 15% in steeping, 55% in germination, and 23% in fermentation. Total phenolics were reduced by 28% for extended steeping, and 45% in germination.	AOA was enhanced due to increase in soluble phenolic compounds during hydration, while prolonged hydration reduced the phenolics in fermentation. Leaching during steeping and phytase activity during fermentation reduced the antinutrients. Loss of total phenolics was due to degradation of phenolics by polyphenol oxidase and peroxidase during germination. Fermentation not only reduces the antinutrients but also reduces the structural and antioxidant properties.	[83]
Germination	Foxtail and proso millet soaked for 15 h, germinated at 24 °C for 4 days. Later, freeze drying and milling and sieving (250 µm).	Decline in tannin (0.33 to 0.02), saponin (0.39 to 0.07), and phytic acid (6.4 to 2.6 mg/g) after germination in finger millet. Similarly, proso millet germination reduced tannins (0.51 to 0.05), saponin (0.44 to 0.07), and phytic acid (8.09 to 4.8 mg/g). The phenolic acids (eight identified) and flavonoids increased by nearly two folds in both millets during germination.	Reduction in tannins was due to their usage as precursors in biosynthesis of different phenolic acids and flavan-3-ols. Saponins reduced due to dissolution in soaking water and grain metabolism. Germination increased phenolic acids and flavonoids due to enzymatic degradation, breakdown of cell wall structure, release of free polyphenols, and conversion of bound form to free phenolics. Prolonged germination affects the accumulation of bioactive compounds.	[84]
Germination	Finger and pearl millet germinated for 48 h at 35 °C, dried at 40 °C, milled, and sieved (100 µm) to produce flour.	Raw pearl millet flour containing 0.61, 0.55, and 15.11 mg/g of tannin, saponin, and phytate reduced to 0.45, 0.96, and 6.34 mg/g, respectively, after germinated flour. In germinated finger millet flour, the tannin reduced (1.61 to 1.04), saponins increased (2.07 to 4.45), and phytates reduced (15.98 to 9.77 mg/g) compared to raw millet flour. Germination increased DPPH FRSA (%) of pearl (55.2 to 82.4%) and finger (71.3 to 80%) millets. Combination of germination and fermentation offered highest increase in DPPH RSA from 18.4 to 33.4%, and FRAP RSA from 10.2 to 13.6 mM TE/g.	Leaching of polyphenols into soaking water and increased enzymatic action reduces tannins. Triggered activation of enzymes, breakdown of complex compounds, and mobilization of stored compounds leads to increase in saponins. Activation of cell-wall-degrading enzymes increases the total phenol. Hydrolysis of phytate phosphorus into inositol monophosphate due to enhanced phytase activity during germination and leaching during hydration reduces the phytates. The free radical scavenging activity is enhanced due to activation of enzymes, fermenting microbes, and bound phenol release during germination. Bioprocessing enhances AOX activity by enzymatic degradation of the cell wall and release of bound bioactive compounds.	[50]
Decortication and polishing	Foxtail and proso millets dehusked in centrifugal sheller and debranned in cone polisher.	Foxtail-total dietary fiber reduced from 20.3–20.8 (whole), to 7.1–7.7 (brown millets), and 3.6–4.3 g/100 g (polished) TPC and TFC reduced from 1.7–0.5 mg/g and 0.7–0.14 mg/g, respectively. Phytic acid reduced from 9.9–4.9 mg/g Proso—total dietary fiber reduced from 19.4 (whole) to 11.1 (brown) and 5.5 g/100 g (polished) TPC and TFC reduced from 1.2–0.24 mg/g and 0.39–0.1 mg/g, respectively. Phytic acid reduced from 7.2–2.2 mg/g	Removal of husk and bran reduces the TDF, TPC, TFC, and phytic acid	[38]
Milling/refining	Pearl millet whole flour, refined, and bran fraction	Phytic acid in milled refined flour was lower (0.6 g/100 g) than whole flour (0.8 g/100 g) Oxalates in whole flour were higher (45 mg/100 g) than the refined flour (35 mg/100 g)	Milling reduces the phytates and oxalates in refined flour due to removal of bran fraction.	[85]
Germination	Finger millet germinated for 12, 24, 48, 72, ands 96 h	TPC and tannin content were reduced from 38.4 to 42.6% and 33.3 to 61.6%, respectively. Flavonoids and AOA increased from 26.6 to 33.3% and 48.3 to 51.1%, respectively. Phytates reduced from 51.67 (non-germinated) to 21.67 mg/100 g Tannins reduced from 53.3 (control) to 23.3 mg/100 g. Trypsin reduced from 0.47 to 0.00 mg/100 g	Reduction in tannins and phytates increases with germination time due to activation of hydrolytic enzymes.	[40]
Germination	Foxtail germinated for 19.5 h up to 46.45 h at various soaking time, ranging from 5.27 to 18.73 h, and germination temperature ranging from 13.18 to 46.8 h.	TPC and TFC increased from 62.45 to 90.54%, 30.52 to 43.96 mg RU/g, and 23.03 to 45.67 mg GAE/100 g, respectively. Tanninand phytate decreased from 2.8 to 0.98 mg/100 g and 0.34 to 0.1 mol/kg, respectively.	The free, bound, and total phenolic compounds are liberated due to the action of cell-wall-degrading enzymes during germination. Antinutrients are reduced due to hydrolytic activity of phytase converting phytate phosphorus into inositol monophosphate.	[86]
Germination & popping	Finger millet germinated for 48 h and popped.	Germinated (360.5) and popped (610) millet flour had less tannins than whole millet flour 870.9 mg/100 g. Germinated (238.5) and popped (333.1) millet flour had less phytic acid than whole millet flour 851.4 mg/100 g. Germinated (29.8) and popped (32.2) millet flour had less oxalates than whole millet flour, 45.8 mg/100 g. Trypsin inhibitor was reduced from 4188 U/g after germination (2001 U/g) and popping (3090 U/g).	Germination and popping significantly reduce the antinutrients and enhance acceptability, digestibility, and bioavailability.	[87]
Soaking, germination, & fermentation.	Foxtail millets soaked for 12 h, germinated for 12 h, fermented for 20 h at 38 °C.	Tannins in untreated sample (86.5) reduced by soaking (73.3), germinating (53.5), fermenting (60.3 mg/100 g). Phytic acid in untreated sample (7.4) reduced by soaking (6.3), germinating (4.5), fermenting (5.2 mg/100 g). Saponins in untreated sample (87.3) reduced after soaking (13.6), fermentation (65.4), and increased after germination (113.2 mg/100 g). Total phenolics in untreated sample (298) reduced by soaking (240), increased by germination (338) and fermentation (328 mg GAE/100 g). Total flavonoids in untreated sample (628) reduced after soaking (619), increased by germinating (658) and fermenting (682 mg QE/100 g). DPPH antioxidant activity was decreased by soaking, while increased by germination and fermentation.	Processing operations significantly alter the antinutrients and bioactive constituents. Soaking leaches antinutrients and water-soluble phenolic compounds. Germination activates phytase enzymes, polyphenols oxidase, and tannins acyl hydrolases during bioprocessing. Saponin reduction during soaking is due to leaching and splitting of saponins by β-glucosidase during fermentation, and their enhancement is due to enhanced biosynthesis.	[26]
Fermentation	Millet matrix was prepared with kodo and proso millet flour + acid whey (variable composition 0-100% *v*/*v*) and homogenized for 3 min.	TPC (mg GAE/g) was found to increase from 0.09 to 0.15 and 0.12 to 0.17 for kodo and proso millet, respectively, with increase in matrix from 25 to 100%. Tannin content of 69.1% in kodo millet and 82.8% in proso millet were reduced. The phytic acid was also reduced by 9.21% for 100% matrix of kodo and acid whey.	Tannin reduction was due to hydrolysis of tannins by tannase. Increase in phytase activity during fermentation reduced the metal chelating activity of phytate and enhanced the bioavailability. LAB can also degrade the phytate by producing the phytase enzyme that hydrolyzes the phytic acids into orthophosphate. LAB dropped because the acid whey serves as enzyme phytase and tannase source.	[88]
Ultrasound assisted sprouting	Sorghum grains sonicated at various amplitudes and times (40% for 5 min, 60% for 5 min, 40% for 10 min, 60% for 10 min) at 35 °C with pulsed on:5 s and pulsed-off 10 s. Treated grains germinated on paper towel for 48 h at 25 °C.	The highest germination (94%) was obtained for 40% amplitude for 5 min treatment, with increase in amplitude and time further reduced the germination linearly. The alkaloids, phytates, saponins, and tannins all reduced from 0.051–0.035, 146–143.2, 0.15–0.09, and 0.14–0.13 mg/100 g, respectively, for 40% amplitude sonication for 5 min, later increases in amplitude and treatment time increased these phytochemicals TPC (mg GAE/g), DPPH (%), and FRAP (mM FE/g) were decreased from 1.18–1.26, 83.7–89.1, and 0.029–0.031, respectively, for 40% amplitude-5 min treatment, and later they were reduced with further increase in amplitude and time. TFC (mf QE/g) was first increased (0.88–1.02) at 40% for 5 min treatment and decreased (1.02–0.78) with sonication amplitude and time increased.	Sonication at 40% amplitude for 5 min was the best treatment to enhance the phytochemical composition. Sonication can be a potential way to enhance the germinated sorghum sprout RSA and phenolic compounds.	[89]
Milling	Proso, Pearl	Dehusking followed by polishing.	TPC decreased from 164.46 to 145.81 mg GAE/100 g (dehusked) to 69.37 mg GAE/100 g (polished grains). TFC reduced from 133.03 (in whole grains) to 119 mg GAE/100 g (dehusked) to 51.23 mg GAE/100 g (polished). Phytate reduced from 574.74 to 194 mg/100 g after polishing. Antioxidant capacity (DPPH) reduced from 32.55% to 16.27% after polishing.	[85,90]
Germination	Foxtail	Germination for 46.5 h. Soaking for 16 h followed by germination at 25 °C for 48 h.	Tannins and phytate decrease with increasing germination time. Showed increase in ferulic, gallic, chlorogenic, sinapic, vanillic acids, and total phenolic acids but caused significant decrease in caffeic, p-hydroxybenzoic, and p-coumaric acid contents.	[86,91]
Germination	Foxtail	High-pressure soaking.	Tannins and phytic acid reduced by 45.07% and 39.02%, respectively.	[59]
Germination	Pearl Variety: Kalukombu (K) and Maharashtra Rabi Bajra (MRB)	Germination for 72 h.	Oxalates reduced up to 24% for K variety and 48% for MRB grains/ Phytic acid reduced from 0.78 to 0.37 g/100 g for K variety and 0.57 to 0.36 g/100 g for MRB variety. Increased the tannin content by 17% as compared to the native grain.	[85,92]
Germination	Kodo	Germination for 13 h.	Reduced tannin content from 1.603 to 0.234 mg/100 g. Phytate content reduced from 1.344 to 0.997 mol/kg.	[93]
Malting	Pearl	Alkaline steeping with (2% Ca(OH2)) and 2% ash solution.	Both solutions resulted in increased level of phytate. Ash steeping increased the level of cyanide (0.03 mg/100 g at 24 h to 0.05 mg/100 g at 72 h). Gradual reduction in tannins and phytate over the period of 24 to 72 h in ash-steeped samples.	[94]
Fermentation	Pearl Var; Sosart-1	Pure cultures of Lactobacillus plantarum.	Phytate content reduced from 2.80 to 0.09%. Tannin reduced from 1.78 to 0.08%	[95]
Fermentation	Proso, Kodo	Fermentation. (Penicillium camembert).	Phytic acid content reduced from 13.43 to 8.53 mg/100 g. 72% reduction of trypsin inhibitor level in fermented proso millet was observed. No significant changes occurred in kodo millet.	[96]

* All bioactive components appearing in the text expressed in grams were measured on dry weight.

**Table 6:** Summary of the effect of thermal processing methods and their influence on antinutritional compounds.

Processing Method	Processing Conditions	Results and Important Observations *	Inference	References
Microwave treatment	Kodo millets were treated at 360, 540, 720 W for 150, 210, and 270 s in domestic microwave oven (2450 MHz).	TPC (mg GAE/100 g) and DPPH AOA (%) of untreated kodo millet were 3.26 and 92.1, respectively. TPC increased (3.29 to 3.42, 3.39 to 3.46, and 3.53 to 4.23) with an increase in treatment duration (150 to 270 s) at all three power levels, 360, 540, and 720 W, respectively. The AOA was found to reduce (91.1–87.9%) with increase in treatment duration and power levels.	During microwave heating, the protein and phenolic compounds change the chemical structure and degrade the hydrolysable tannins and phenolic compound, which increases TPC.	[97]
Parboiling, decortication.	Pearl and proso millets were parboiled as follows: soaking for 12 h at 30 °C, boiled at 100 °C for 5 min, and dried for 24–48 h until final moisture of 11–13%. Decortication using abrasive mill at 1400 rpm for 2 min to remove pericarp and germ.	Porridge and couscous products were prepared using decorticated parboiled and non-parboiled millets. Porridge involves grinding millet in flour mill and cooking in boiling water for 10 min. Couscous involved millet soaking for 1 h, steaming for 30 min. Free phenolics (µg/g) in native pearl millet’s porridge (25) and couscous (71.4) increased to 74.8 and 117 for parboiled porridge and couscous, respectively. Maximum increase in free phenolics was observed in porridge made by parboiled proso millet. Parboiling significantly increased the concentration and formation of phenolic acids in both proso and pearl millets. DPPH FRSA of pearl millet products extracted from native millet (13.2–27.6%) was increased by parboiling (25–35.5%).	Decortication reduced phenolic content due to removal of outer layers. Parboiling enhanced the free phenolic in porridge and couscous by 200 and 64% and bound phenolics by 41 and 47%, respectively. Pearl millet products had better AOC compared to proso millets due to their high phenolic profile. DPPH RSA of free extract of proso and pearl millet couscous was higher than porridge. Parboiling increases decortication yield and also enhances the phenolic acids and AOA.	[98]
Roasting and fermentation.	Finger millet rinsed in water, dried, and roasted on open pan for 10–15 min. Fermentation: millet was soaked under covered sack and fermented for 24 h and sun dried.	Fermented millets had flavonoids, terpenoids, tannins, and saponins while roasted millets had alkaloids, terpenoids, and tannins. DPPH RSA of fermented millet was 26.4% less than both roasted and unprocessed millet (31.8%).	Saponin absence in roasted millet was due to processing method and high temperature used. The reduction in AOA was due to loss of phenolic acids and flavonoids during fermentation. Fermentation enhanced Ca, Ph, K, and Fe minerals, except Mn and Zn. Roasting enhanced Mn and Zn	[99]
Parboiling, and decortication.	Pearl millets were processed as follows: soaked for 4 h with increasing temperature (60, 70, and 80 °C), cooled, sun dried for 1 h, steamed again using parboiling system for 15 min, sun drying (1 h) until 10–12% final moisture, milling in abrasive disc huller with residence time of 3 min to decorticate grains.	Phytate (mg/100 g) in un-parboiled undecorticated millet (with husk) ranging from 981–928 was reduced after decortication to 785, further reduced to 483–656.5 after parboiling at various temperatures. There was no significant difference in reduction due to parboiling temperature (60 and 70 °C).	Phytic acids varied with cultivars, dehulling removes seed coat, pericarp, and aleurone layers containing phytates and polyphenols. Removal of bran drastically reduces the antinutrients. Optimized soaking temperature (80°C) had a strong influence on phytate reduction (by 20–30%), which was due to leaching.	[100]
Roasting and grinding	Finger millet roasted and grinded in a home blender (conditions unspecified).	The phenols (mg/100 g) in unroasted (314.2) was higher than roasted (223.3) millet flour. Roasting also reduced the antioxidant activity from 89.8 to 86.7%.	Roasting and grinding processes lead to digestible enhancement and thermal degradation of phenolic and flavonoids, which reduced AOA.	[101]
Microwave and steam treatments	Pearl millets subjected to microwave (40, 60, 80, and 100 s) and soaked grains (65 °C for 2 h), steaming under pressure (4, 6, 8, and 10 min) treatments.	TPC (mgGAE/100 g) of untreated was linearly reduced from (234.4 to 175.5 with microwave duration increase (0–100 s). TPC (mgGAE/100 g) of untreated (234.4) was also reduced (148) with steaming duration (0–10 min).	Hydrothermal treatment of pearl millet reduced TPC without altering the minerals. Bioaccessibility of Fe and Zn were enhanced after hydrothermal treatments.	[102]
Roasting, steaming, puffing, and extrusion.	Boomcorn millet: Dehulled to remove 98% hull. Roasting: 110 °C for 10 min after soaking for 6 h and milled (200 µm sieve). Steaming: 110 °C for 10 min, oven dried at 50 °C, and milled. Puffing: 110 °C at 1 MPa and milled. Extrusion: Twins screw extruder, feed rate (30 g/min), 150 rpm, 110 °C with 80–100 bar pressure. Oven drying of extruded samples at 50 °C, milled.	Total phenols (670 mg/100 g) and total flavonoids (391 mg/100 g) were highest in roasted > puffed (TP: 645 mg/100 g, TF: 304 mg/100 g), >extruded (TP: 455 mg/100 g, TF: 219 mg/100 g), and >steamed (TP: 315 mg/100 g, TF:282 mg/100 g) samples. The TPC and TFC in dehulled millets was lesser than whole millets following the order: roasted > puffed > extruded > steamed > unprocessed for TPC and roasted > puffed > steamed > extruded > unprocessed for TFC. AOA of different processing methods followed the order: roasted > puffed > steamed > extruded > unprocessed sample for both dehulled and whole millets.	Whole roasted millet accumulates higher phenolic acids. Processing methods enhance the accumulation of secondary metabolites and antioxidant compounds. Heat treatments induce the hydrolysis of c-glycosylflavones and release free phenolics. Bioaccessibility of TFC is more in processed millet, and it was 2-folds more in roasted sample than unprocessed millet. Increase in AOA in processed samples was due to the increase in TPC and TFC.	[103]
Microwave processing of sprouted grains	Sorghum grains soaked overnight, microwave treated at 450 W for 15–30 s, and 700 W for 15–30 s, and germinated at 25C for 48 h.	Optimized treatment with best results was 700 W for 15 s. Microwave treatment enhanced germination while reducing the saponins and phytate, but not tannins and alkaloids. Alkaloids (0.051 mg/100 g), saponins (0.015 mg/100 g), tannins (0.147 CE/100 g), and phytates (146 SPE/100 g) varied from 0.03–0.05, 0.07–0.017, 0.12–0.149, and 142–150, respectively, for microwaved sprouted grains. Highest TFC (1.05 mgQE/g), TPC (1.27 mg GAE/g), DPPH (89.7%), and FRAP (0.03 mM FE/g) were recorded for 700 W for 15 s treatment as compared to control.	Alkaloids are lost during soaking, solubilization and leaching reduced the saponins, leaching and hydrophobic association of tannins with enzymes and proteins reduced tannins, and phytase activity enhanced the phytates. Biochemical reactions during germination significantly change the metabolites, phenolic profile, and AOA.	[104]
Hydrothermal, microwave, and infrared treatments.	Pearl millet flour (0.3 mm) sieved and microwave treated (MW) (750 W for 90 s). Millet flour was treated with infrared (IR) radiation with 150 W, 0.7–1 µm wavelength at 70–80 °C for 5 min. Hydrothermal treatment (HT) was done soaking millet grains for 1 h, milled to paste, and steam treated in a cloth at 100 °C for 5 min, later incubated and dried overnight at 50 °C and milled into flour.	A significant reduction (10.6%) in bioaccessibility of phenolics was observed for hydrothermal treated (HT) samples compared to native meat flour roti. Pearl millet flour roti (India bread) was prepared by baking on an iron griddle at 200C for 50C. The TPC (mg GAE/g) of 5.56 in native flour was reduced to 4.98 (HT) and increased to 6.06 (MW) and 6.3 (IR). The bioaccessibility (intestinal digestion) of Fe and Zn was reduced by HT, while changes under IR and MW treatments was not significant. The FRAP AOC (µM/g) was highest in IR: 2.08, followed by MW: 2.06, HT: 1.78, and native millet flour: 1.91.	Hydrothermal treatment was a promising method to enhance the nutrient value and reduce the glycemic index and enzymic activity related to rancidity. HT treatment reduced the bioaccessibility of phenolics (10.6%) and minerals (2–3.2%) compared to the MW and IR treatments.	[24]
Roasting	Barnyard millets roasted at 70–80 °C for 6–7 min.	Tannins and phenolic compounds reduced from 1.36 to 1.02 mg/100 g and 4.31 to 3.2 mg/100 g, respectively.	Roasting reduced the antinutrients mainly due to heat degradation and slightly due to evaporation of these compounds during roasting.	[51]
Dehulling, roasting, cooking.	Barnyard millet dehulled in mortar, roasted till golden brown, and boiled in water until cooked. (Unspecified conditions).	TPC (1.59) in raw millet reduced to 0.31, 0.68, and 0.25 g/mg after dehulling, roasting, and boiling. TFC was also reduced from 3.51 to 1.21, 0.68, and 0.16 mg/g, respectively, post-processing. The DPPH and FRAP activity were lower in dehulled (0.07, 0.78), roasted (0.25, 0.85), and boiled samples (0.21, 0.28) compared to raw millet (0.28, 3.08 mg AAE/g DW). Oxalates (0.12%) and phytic acid (4.86%) were also reduced by dehulling (0.105, 3.18%), roasting (0.038, 3.16%), and boiling (0.023, 3.19%), respectively.	The phenolics bind with other molecules like proteins, leading to reduction in extractable TPC and TFC after heat processing. Dehulling, roasting, and boiling reduces FRAP activity by 74, 72, and 90.0% and DPPH activity by 75, 10.7, and 25%, respectively, due to reduction in phenolic compounds during heat processing. Thermal processing reduces the oxalates and phytic acids due to heat degradation.	[105]
Steaming, pressure cooking, dry roasting, open boiling.	Finger millets were used to develop various products with different processes Roasting on hot pan for 10 min (Unfermented bread/rotti), steaming for 15 min (pittu), pressure cooking for 15 min (halape), and open boiling for 25 min (thalapa-thin porridge). Grains were dehulled (rice polisher) and ground (0.038 mm), then boiled for 20 min to prepare thin porridge.	TPC of soluble and bound phenolics ranged from 29.6 to 79.6 and 2.2 to 11.8 µM FAE/g, respectively. TPC was in the following order: porridge > thlapa > hapale > pittu > rotti. Pressure cooking (33%), boiling (67%), and roasting (17%) increased and boiling (12–19%) reduced soluble phenolics. Soluble flavonoids (µM CE/g) reduced by 26, 23, 13%, respectively, in pressure cooking, roasting, and steaming, while increased by 0.5–1.5 folds in open boiling. TEAC-Trolox equivalent antioxidant capacity (µM TE/g) of soluble and bound phenolics was in the range of 0.9–5.2 and 0.7–0.38, respectively. Highest DPPH FRSA was for boiled food, and 2, 2.2, 1.6, and 1.4 time lesser than pan-roasted, steamed, pressure-cooked, and open-boiled products, respectively.	Hydrothermal treatments are effective in altering the TPC due to release of bound phenolics. Release of bound phenolics, condensation, oxidation of phenolics, thermal degradation, Maillard reaction, and depolymerization are the reasons for change in phenolic compounds and their respective AOCs.	[92]
Hydrothermal treatment and milling	Finger millet soaked (10 h) at 30 °C, steamed (98 kPa for 20 min), and try dried (39 °C till 13% moisture content). Milling at variable roller speed (1000, 1200, and 1400 rpm), residence time (10, 12, and 14 min), and dispersed density * 430, 490, 560 kg·m^3^).	Reduction of tannins (10.3–50%) was noted due to combined effect of pearling and hydrothermal treatment. Increase in residence time (12–14 min) had maximum reduction in tannins (2.5–3.5 ppm). Under optimum milling conditions of roller speed (1001.3 rpm), residence time (13.9 min), dispersed density (560 kg·m^3^) with a 7.4% degree of pearling, the tannin content (3.19 ppm) was lower than unprocessed millet (4.14 ppm).	Hydrothermal treatment and pearling reduce the tannins slightly while also reducing nutritive value. The loss of tannins was attributed to the loss of bran layer during pearling, indicating the accumulation of tannins in outer layers. Models developed in the study could be used to predict the loss of nutritive and antinutritional values as a function of different variables studied.	[106]
Germination and roasting	Jirani millet germinated (24–72 h) and roasted at 112.5–120C for 15–21 min.	AOX capacity ranged from 39.3–66 mg/100 g, DPPH from 68.2–79.6 µg/mL, and reducing power from 0.35–0.44 µg/mL for germinated and roasted sample. Optimized conditions were 68.9 h (germination time), 114.7 °C (roasting temperature), and 15 min (roasting time), resulting in 54.6 mg/100 g (AOC), 72.1 µg/mL (DPPH), and 0.37 µg/mL (reducing power).	Germinated and roasted millet may be used as an ingredient to formulate functional food.	[107]
Blanching	Pearl millet blanched in boiling water at 98 °C for 30 °C and dried at 50 °C for 60 min.	Blanched pearl millet had less TPC (1.19 mg GAE/g) and DPPH FRSA (35.6%) compared to unblanched millet (1.29, 38.5%, respectively).	The water blanching reduces the TOC and AOA, but not significantly. Blanching improved the mineral contents of food grains studied.	[49]
Cooking, fermentation, and Baking	Foxtail and barnyard millet moistened (10%), hand pounded to dehull, and dried. Cooking: soaking (3, 6, and 12 h) and cooking (19, 22, and 25 min). Fermentation: dehulled grains soaked for 10 h at 28C, wet grinded, and fermented overnight. Baking: millet flour mixed with other ingredients, baked at 180 °C for 25–30 min.	Formulated fermented and baked products. Foxtail and baranyard millets had 0.6 and 0.22 mg GAE/g (TPC), 28.9 and 7.61 mg QE/g (TFC), and 12.9 and 6.6 mg TAE/g (total tannin content), respectively.	The TPC, TPC, and TTN contents of cooked, fermented, and baked products are analyzed in this study. The effect of processing methods on phenolics and antinutrients could not be comprehended.	[108]
Baking	Germinated finger millet (GFM) and germinated bambara ground nut (GBGN) composite flour used in 100:0, 90:10, 80:20, 70:30, and 60:40 ratios baked at 180 °C for 20 min.	In 100% GFM flour baked biscuits, the phytic acid decreased (3.24–2.41 mg/g), TPC increased (1.64–1.89 mg GAE/g), FRAP increased (4.13–4.17 µM TE/g), and ABTS reduced (2.5–2.12 µM TE/g) after baking.	Phytic acid of biscuits reduced as %GBGN increased in the flour blend. No trypsin inhibitor was detected in biscuits with 100% GFM. Thermal degradation during baking reduces the antinutrients and increases digestibility and bioavailability of minerals. Increased FRAP and ABTS values were due to higher synthesis of phenolic compounds and GBGN flour’s high antioxidant potential.	[109]
Roasting and germination	White finger millets pan roasted (120 °C for 5 min) and germinated (30 °C for 48 h). Dried and milled with sieve size < 250 µm.	The highest TPC (mg GAE/100 g) and PFC (mg QE/100 g) observed in roasted (0.11, 0.84), followed by native (0.04, 0.73) and germinated flour (0.02, 0.641). DPPH AOA of native (51.5%) increased to 57.53% (roasting) and 77.9% (germinated flour).	Germination and roasting have contrary effects on TPC and TFC. TPC and TFC increase during germination was due to loss of hydrolyzed phenolic compounds. Maillard reaction can enhance the TPC during roasting. The interaction between total phenols and organic compounds and minerals may reduce AOA during germination, and formation of non-enzymatic browning compounds may enhance the AOA during roasting.	[110]
Extrusion	Finger millet flour with 20% moisture extruded at 110 °C and 350 rpm to develop composite bread.	The bread with extruded millet flour had highest TPC (80.7 mg FE/100 g) and AOX (10.7 mM TE/100 g dw) activity, followed by bread with unextruded millet flour (TPC-50.3, AOX-10.8) and control (TPC-20, AOX-9).	High-temperature extrusion breaks down the cell wall, releases bound phenolics, and initiates Maillard reaction, resulting in increase in phenolic content and AOX activity.	[111]
Extrusion	Proso millet extruded at various moisture (17–25%), screw speed (170–250 rpm), and temperature (90–150 °C)	Antioxidant activity increased with increased screw speed and decreased as moisture and temperature increased. Antioxidant activity extruded millet ranged between 16.5 and 31.4 mM/g, and was significantly affected by temperature.	High shear rates resulted in breakdown of cell wall complex to release more phenolics. Maillard reaction also increases the antioxidant activity.	[112]
Cooking	Pearl	Blanching 98 °C for 10–20 s. Dry heat treatment (roasting).	Reduced levels of polyphenols and phytic acid contents. Substantial reduction in tannins.	[113]

* All bioactive components appearing in the text expressed in grams were measured on dry weight basis.

**Table 7:** Summary of the effect of non-thermal processing methods and their influence on antinutritional compounds.

Processing Method	Processing Conditions	Results and Important Observations *	Inference	References
γ-Irradiation	Finger millet slurry (CO-14 and CO-15 varieties) γ-radiation source: cobalt-60 Dose: 2.5, 5, 7.5, and 10 kGy Dosage rate: 2.5 kGy/h	Native Finger millet flour had a phenolic content of 166.05 mg/100 g (CO14) and 175.64 mg/100 g (CO15), which decreased to 126.68 mg/100 g and 124.03 mg/100 g after 10 kGy irradiation. Insignificant increase (*p* > 0.05) in total flavonoids on increasing dosage, recorded 122.49 and 118.72 mg/100 g on 10 kGy irradiation as compared to 98.16 (CO-14) and 99.56 mg/100 g (CO-15) in the native flour. Tannins reduced from 249.71 and 249.72 mg/100 g to 192.98 and 192.06 mg/100 g on increasing dose to 10 kGy. Increment in dosage, reduced phenols, and tannins but increased flavonoids.	The reason for the decrease in phenolics is unclear but may be a result of the decomposition of insoluble phenolic complexes, analogous to findings of other studies. The increase in flavonoids is attributed to the degradation of other molecules previously bound to flavonoids thereby increasing its availability. Decrease in tannins is observed owing to degradation of protein complexes and increase in solubility of tannins	[114]
γ-Irradiation Electron beam Infrared Radiation	Sorghum grains γ-Irradiation: Co_60_ at 10, 20 and 30 kGy doses. Electron beam: Rhodotron accelerator model TT 200 at 10, 20 and 30 kGy doses. Infrared Radiation: 1000 W IR at time intervals of 60, 90 and 120 s.	Reduction in phenols from 1155 to 210 mg catechin equivalent/100 g dry matter on exposure to 30 kGy. Tannins progressively reduced from 921 mg catechin equivalent/100 g dry matter in control to 488, 409 and 191 mg catechin equivalent/100 g dry matter after being treated at 10, 20 and 30 kGy. Phytates decreased from 774 to 147 mg/100 g dry matter on gamma irradiation at 30 kGy. Electron beam reduced phenols, tannins and phytates from 1155, 921 and 91 to 253 mg catechin equivalent/100 g dry matter, 242 mg catechin equivalent/100 g dry matter and 91 mg/100 g, respectively. Electron beam was more efficient in reducing phytates at the same dose as compared to gamma irradiation. However, the opposite was true for tannins and phenols, where gamma irradiation was more potent. IR reduced phenols, except that the treatment at 120 s increased phenol content to 1228 from 1171 mg catechin equivalent/100 g at 90 s. A general decrease in tannin content from 921 to 419 mg catechin equivalent/100 g at maximum treatment time 120 s. Progressive reduction in phytates to 456, 201 and 67 at 60 s, 90 s and 120 s.	Irradiation reduced phenolics due to accelerated formation of protein phenols complex that reduced free phenols. Phenols decreased under IR owing to thermal alterations. Tannins reduced due to the formation of OH- and superoxide radicals. The mechanism of electron beam in reducing tannin is unavailable. IR is believed to alter the solubility and reactivity of tannins Phytate reduction due to cleavage by free radicals produced during gamma-irradiation. Phytate reduction by electron beam unclear. Infrared decomposed phytates to inositol and lower phosphates.	[115]
Ozone	Foxtail millet Ozone dose: 0.06 L/min Treatment time: 15, 30, 45 and 60 min	Decrease in tannins from 112.43 to 64.83 mg TA/100 g, however, increase in phytic acid from 8.15 to 9.75 mg PA/g. Decrease in TFC 331.46 to 273.59 mg QE/100 g. Increase in TPC from 132.93 to 196.66 mg GAE/100 g. A decrease in antioxidant activity was noted.	Ozone induced oxidation reduced flavonoids. Ozone inhibits the enzyme activity of peroxidase and PPO, thus preventing the loss of phenols. Decrease in tannin content is due to structural alterations of carboxyl or carbonyl groups in tannins that affect its activity. Increase in phytates is due to increased surface area and gas exposure associated.	[116]
Ozone	Red sorghum flour Ozone dose: 32.0, 38.0, 44.0 and 50.0 g/kg	Ozonation led to an increase in total phenolic content (from 226.34 to 276.23 mg GAE/100 g) and FRAP activity (from 8.14 to 12.65 µmol TE/g). It decreased total flavonoid content (from 605.26 to 582.15 mg QE/100 g) and DPPH activity (from 10.90 to 9.61% RSA).	Ozone reduces TFC due to oxidation of flavonoids. While increase in TPC is due to protection offered from phenol degrading enzymes.	[117]
Atmospheric Cold Plasma (ACP)	Pearl millet Power level: 20 kV; 25 kV; 30 kV Treatment time: 10 min, 15 min; 20 min	Maximum reduction in tannin and phyate was observed at 30 kV; 20 min. The control samples, initially containing 0.98 g of tannic acid per 100 g dry matter and 9.25 g of phytate per 100 g dry matter, were reduced to 0.81 g and 6.71 g, respectively. TPC, TFC and AOA decreased (*p* < 0.05) on increasing power/duration of cold plasma.	ROS (Reactive Oxygen Species) act on the glycosidic bonds of tannins, while free radicals generated during cold plasma treatment cleave phytate rings. ACP forms ROS an ozone that degrades the phenolic ring and forms aliphatic compounds. This reduces TPC, TFC and antioxidant capacity due to loss of the aromatic ring.	[118]
Cold plasma	Foxtail millet Power level: 1 kV, 2 kV Treatment time: 1, 3, 5 min	Tannin and phytate was reduced to 0.8 mg/100 g and 1.1 mg/100 g.	The treatment with cold plasma increased the germination percentage which reduced tannin and phytate due to the action of phytase enzyme.	[119]
Cold plasma	Pearl millet; Barnyard millet Power: 2 kV Treatment time: 5 min	Pearl millet’s TPC decreased from 84.03 to 82.28 mg/100 g, and its TFC decreased from 303.21 to 271.1 mg/100 g after cold plasma treatment. Barnyard millet’s TPC decreased from 57.71 to 46.60 mg/100 g, and its TFC decreased from 371.64 to 344.73 mg/100 g following treatment with cold plasma. 18% and 24% reduction in tannin content of pearl and barnyard millet on exposure to cold plasma. 57% and 61% decrease in phytates of pearl and barnyard millet, respectively. Antioxidant activity decreased in pearl and barnyard millet on treatment with cold plasma.	Obliteration of flavonoids by ROS and decomposition of aromatic rings of phenols to aliphatic forms. The free radical generated during cold plasma treatment breaks glycosidic bonds in tannins and interferes with phytate rings. Antioxidant activity decreased owing to the neutralizing effect of radicals formed during cold plasma and the reduction in phenol and flavonoids.	[120]
Multipin atmospheric cold plasma (MACP)	Barnyard millet flour Power: 10, 20, 30 kV Treatment time: 10 min, 20 min, 30 min	TPC: Increased from 229.33 to 280.54 mg GAE/100 g after 30 kV for 30 min. TFC: Increased from 173.75 to 244.71 mg QE/100 g after 30 kV for 30 min. Tannin content: Decreased from 238.04 to 174.53 mg/100 g after 30 kV for 30 min. Phytic acid content: Decreased from 387.02 to 321.31 mg/100 g after 30 kV for 20 min.	An increase in treatment time and voltage enhanced the cleavage of covalent bonds with bigger molecules that liberated phenols and flavonoids. Additionally, the chemical species disrupt cell membranes increasing the extractability of bioactive compounds. Tannins and phytates reduced due to disruption of glycosidic bonds and phyatete rings. Conversion of antinutrients into insoluble forms by free radicals.	[121]
High pressure	Foxtail millet germinated and non-germinated Pressure: 200, 400 and 600 MPa Temperature: 20, 40, 60 and 80 °C Treatment time: 30, 60, 90 and 120 min	The tannin and phytic acid in the control germinated foxtail millet flour reduced from 0.5421% to 0.1870% and from 0.0016% to 0.0006% after treatment at 600 MPa and 60 °C for 120 min. TPC and antioxidant activity of foxtail millet flour increased on increasing pressure, time and temperature.	Increased solubility of phytate cation complexes and tannins that are migrated under pressure. Increased pressure on cell walls releases bound phenols and degrades cell walls.	[59]
Ultrasound	Sorghum Amplitude: 40%; 60% Duration: 5 min; 10 min. Frequency: 20 kHz, Power: 750 W Pulsed on/pulsed off time: 5 and 10 s. Temperature (constant): 35 °C	Optimal condition was determined at 40% for 5 min where increment for TPC (mg GAE/g) 1.18 → 1.26, DPPH (%) 83.7 → 89.1 and FRAP (mM FE/g) 0.029 → 0.031 was observed. Increase in amplitude and duration of treatment reduced TPC. The control group had alkaloid levels of 0.051 mg/100 g, phytates at 146.05 mg SPE/100 g DM, saponins at 0.15 mg/100 g and tannins at 0.147 mg CE/100 g DM. Alkaloids, phytates, saponins, and tannins decreased at 40%, 5 min and 60%, 5 min as compared to control but rose with increased treatment time (40%, 10 min and 60%, 10 min). Finally, the US4 treatment (60% for 10 min) significantly increased alkaloids to 0.062 mg/100 g, phytates to 154.46 mg SPE/100 g DM, saponins to 0.17 mg/100 g, and tannins to 0.162 mg CE/100 g DM compared to the control and other treatments.	Increase in TPC, DPPH and FRAP values at 40% and 5 min is due to the action of endogenous enzymes that are released during germination. Germination percentage was the highest for 40%, 5 min combination, as the ultrasound treatment was adequate to cause cavitation that aids in water uptake facilitating germination. Greater time an amplitude may damage cell wall. Germination is correlated with a decrease in saponins, alkaloids, tannins and phytates due to leaching, solubilization and enzyme activity.	[89]
Ultrasound	Kodo millet flour; Little millet flour Ultrasonication time: 10–30 min Germination time: 12–72 h Temperature: 20–40 °C	The study demonstrated that under optimized conditions were ultrasonication (30 min), germination (72 h), and temperature (40 °C) which are the maximum values in the chosen treatment conditions. Kodo millet showed reduced phytate content (0.165 mol/kg) and tannin content (2.88 mol/kg), along with the highest antioxidant activity (88.46% RSA). Similarly, little millet exhibited reduced phytate content (0.199 mol/kg), higher tannin content (9.51 mol/kg), and the highest antioxidant activity (89.06% RSA) at the optimized conditions.	Increase in time and temperature during germination reduces tannin due to leaching and oxidation. Similarly, an increase in ultrasound produces hydroxyl ions that convert tannins to gallic acids. Decrease in phytate, is attributed to increase in phytase activity due to germination and ultrasound treatment. Increase in antioxidants due to cavitation that helps in release of bound phenols and improves their extractability.	[34]
Ultrasound	Finger millet Ultrasound amplitude: 30–70% Pulse on/off time: 5 s Treatment/hydration time: 10–30 min	Increase in amplitude and treatment time decreased TPC, tannins, and phytate. At 65.73%, 26.13 min, 1:3 grain-water ratio, the ultrasound treatment achieved a 66.98% reduction in phytate and a 62.83% reduction in tannin.	TPC decreased by the loss of phenols into the solution as a result of cavitation and increased penetrability. Tannins reduced due to ester link breakage by hydroxyl ions formed during hemolysis of water during ultrasound. Thermal degradation of phytate to inositol reduced as amplitude and time increased, owing to greater heat generation.	[74]

* All bioactive components appearing in the text expressed in grams were measured on dry weight basis.

## Data Availability

No new data were created or analyzed in this study. Data sharing is not applicable to this article.
